# Role of Calcium Signaling Pathway-Related Gene Regulatory Networks in Ischemic Stroke Based on Multiple WGCNA and Single-Cell Analysis

**DOI:** 10.1155/2021/8060477

**Published:** 2021-12-26

**Authors:** Weiwei Lin, Yangxin Wang, Yisheng Chen, Qiangwei Wang, Zhaowen Gu, Yongjian Zhu

**Affiliations:** ^1^Department of Neurosurgery, Second Affiliated Hospital of Zhejiang University School of Medicine, Zhejiang University, 88 Jiefang Road, Hangzhou, 310009 Zhejiang, China; ^2^Department of Orthopedic Surgery, Second Affiliated Hospital of Zhejiang University School of Medicine, Zhejiang University, 88 Jiefang Road, Hangzhou, 310009 Zhejiang, China; ^3^Department of Sports Medicine, Huashan Hospital, Fudan University, Shanghai, China

## Abstract

**Background:**

This study is aimed at investigating the changes in relevant pathways and the differential expression of related gene expression after ischemic stroke (IS) at the single-cell level using multiple weighted gene coexpression network analysis (WGCNA) and single-cell analysis.

**Methods:**

The transcriptome expression datasets of IS samples and single-cell RNA sequencing (scRNA-seq) profiles of cerebrovascular tissues were obtained by searching the Gene Expression Omnibus (GEO) database. First, gene pathway scoring was calculated via gene set variation analysis (GSVA) and was imported into multiple WGCNA to acquire key pathways and pathway-related hub genes. Furthermore, SCENIC was used to identify transcription factors (TFs) regulating these core genes using scRNA-seq data. Finally, the pseudotemporal trajectory analysis was used to analyse the role of these TFs on various cell types under hypoxic and normoxic conditions.

**Results:**

The scores of 186 KEGG pathways were obtained via GSVA using microarray expression profiles of 40 specimens. WGCNA of the KEGG pathways revealed the two following pathways: calcium signaling pathway and neuroactive ligand-receptor interaction pathways. Subsequently, WGCNA of the gene expression matrix of the samples revealed the calcium signaling pathway-related genes (*AC079305.10*, *BCL10*, *BCL2A1*, *BRE-AS1*, *DYNLL2*, *EREG*, and *PTGS2*) that were identified as core genes via correlation analysis. Furthermore, SCENIC and pseudotemporal analysis revealed *JUN*, *IRF9*, *ETV5*, and *PPARA* score gene-related TFs. *Jun* was found to be associated with hypoxia in endothelial cells, whereas *Irf9* and *Etv5* were identified as astrocyte-specific TFs associated with oxygen concentration in the mouse cerebral cortex.

**Conclusions:**

Calcium signaling pathway-related genes (*AC079305*.10, *BCL10*, *BCL2A1*, *BRE-AS1*, *DYNLL2*, *EREG*, and *PTGS2*) and TFs (*JUN*, *IRF9*, *ETV5*, and *PPARA*) were identified to play a key role in IS. This study provides a new perspective and basis for investigating the pathogenesis of IS and developing new therapeutic approaches.

## 1. Introduction

Ischemic stroke (IS) is the most prevalent type of stroke, accounting for 87% of all strokes [1–3]. Because no effective treatment other than thrombolytic therapy is available for the neurological impairment caused by IS, developing new treatments for IS to improve prognosis is necessary.

Investigating signaling pathways involved in the poststroke period may help to identify new approaches to deal with the complex sequelae of IS. Gene set variation analysis (GSVA) is an approach that allows the assessment of potential changes in pathway activity [4]. It has recently been used in studies on pancreatic and breast cancers and has demonstrated excellent potential for identifying prognosis-related pathways in various cancers [5–9]. However, it has not yet been used in stroke research. In addition, microarray technology is effective in screening for pathways and functional genes associated with stroke, and weighted gene coexpression network analysis (WGCNA) can be used to construct coexpression networks between genes and pathways to predict changes in related signaling pathways after IS [10].

We quantified the activity of each pathway in samples using GSVA. Subsequently, WGCNA was used to assess the core genes associated with these pathways and analyse changes in the expression of core genes in these pathways in peripheral whole blood samples after acute IS. Single-cell regulatory network inference and clustering (SCENIC) is used to analyse transcription factors (TFs) for identifying gene regulatory networks (GRNs). We used SCENIC to assess TFs regulating the core genes and their role in cell trajectory development using pseudotemporal and RNA velocity analyses. This study is aimed at assessing the core pathways and their associated genes in peripheral blood samples after IS and revealing the role of TFs targeting core genes at the cell level under hypoxic and normoxic conditions.

## 2. Materials and Methods

### 2.1. Data Acquisition and Processing

The Gene Expression Omnibus (GEO) database (http://www.ncbi.nlm. http://nih.gov/geo/) was accessed via the National Center for Biotechnology Information to search the term “ischemic stroke”. The gene expression profile microarray data for IS were retrieved using the same term.

The inclusion and exclusion criteria were as follows: (i) datasets must comprise genome-wide expression mRNA microarray data, (ii) data should include peripheral blood specimens from both patients with IS and healthy subjects, and (iii) the number of specimens in each dataset must be greater than 20. A total of 40 samples from GSE22255 were retrieved based on these criteria [11]. In addition, GSE110993 was selected as the IS miRNA database for screening IS-associated miRNAs [12].

Based on a search of cerebrovascular-related databases, a total of 3186 mouse cerebrovascular cell samples were obtained from GSE98816 [13]. In addition, a total of 7925 cerebral cortex cell samples from GSE125708 were used to investigate differences in gene transcriptional regulation between the hypoxia and normoxia states [14]. Furthermore, these data were used for single-cell analysis.

### 2.2. Identification of Key Modules

GSVA allows the assessment of potential changes in pathway activity in each sample [4]. The GSVA package in R software was used for the analysis, and the enrichment scores of pathways in all samples were calculated.

The “WGCNA” R package was used to screen for IS-related pathways and genes in the dataset obtained from GSVA according to the following requirements: (i) outlier removal: pathways/genes were clustered hierarchically according to pathway/gene expression patterns, with outliers being removed; (ii) pathway/gene module formation: genes were grouped by K-means clustering to form modules; and (iii) module screening: modules were subjected to principal component analysis. Pearson correlation analysis was performed to derive the relationship between the first principal component of each module and clinical phenotypes. Subsequently, it was screened for pathways/genes that were substantially associated with poststroke development. Pearson correlation coefficient (CC) was used to determine the association between each module and characteristics.

### 2.3. Construction of miRNA-mRNA and Protein-Protein Interaction Networks

The miRWalk 3.0 and miRTarBase database were used to assess the correlation between mRNAs and miRNAs [15]. Genes obtained from WGCNA and differentially expressed genes (DEGs) between patients with IS and healthy controls were entered into a search tool on the STRING to retrieve interacting genes to identify hub genes [16]. SCENIC and TRRUST (version 2) [17] were used to predict the regulatory relationship between TFs and core genes. Cytoscape (v. 3.8.0) was used to visualise the networks.

### 2.4. Single-Cell Analysis

Single-cell analysis was used to validate and assess the expression of hub genes at the single-cell level. Quality control, dimensional reduction, and clustering of the scRNA-seq data of the mouse brain vasculature (GSE98816) were performed using Seurat (v. 4.0.4) as described in a previous study [18]. We annotated clusters using singleR (v. 1.0) followed by manual correction using CellMarker [19, 20].

We further analysed the expression of hub genes obtained from WGCNA in each cell subset using two mouse cerebral samples under hypoxic and normoxic conditions. The scRNA-seq data of mouse cerebral cortices were filtered according to the following criteria: genes expressed in at least 40 cells and cells in which at least 200 genes were expressed. Gene expression was normalised logarithmically and scaled. Variable genes for each of the two samples were calculated using the “FindVariableGenes” function in Seurat (v. 2.2.0). The union of the top 1000 genes with the highest dispersion in each of the two samples was selected as a global set of variable genes for clustering. Subsequently, a canonical correlation analysis was performed to identify common sources of variation between the normoxia and hypoxia groups using the “RunCCA” function. We selected CC1–20 to align the CCA subspaces for clustering based on the correlation strength of each CC using “MetageneBicorPlot” function. We used marker genes reported in the original study to annotate the cell clusters as specific cell type. The Tabula Muris consortium set was also used to annotate the cells using scmap [21]. The integrated scRNA-seq profile was used as the input file of pySCENIC workflow. We used DESeq2 (v. 1.26.0) [22] to identify DEGs in mouse brain samples under normoxic and hypoxic conditions in each cluster.

### 2.5. Gene Regulatory Networks and Hub TFs

A modified SCENIC method (pySCENIC, v. 11.2) was used as previously described to identify GRNs from single-cell transcripts [23, 24]. We identified the key TFs in two steps. The first step was to predict GRNs and TFs using SCENIC, which consisted of the following three steps: establishment of coexpression modules, identification of direct relationship using motif analysis, and calculation of regulon activity score (RAS) with AUCell [18]. We identified the corresponding GRNs and TFs from mouse cerebrovascular cells and cerebral cortex cell samples. In the second step, hub genes obtained from the WGCNA were used to interfere with the hub TFs and corresponding targeted genes in GRNs. Finally, we performed intersection and union analyses of the TF-target gene pairs between GSE98816 and GSE125708. TFs in the intersection analysis were named hub TFs, and those in the union analysis were named key TFs. These TFs were used for further analysis.

### 2.6. Pseudotemporal Trajectory Inference and RNA Velocity

We performed pseudotemporal trajectory inference analysis on the mouse brain vasculature and cerebral cortex using monocle3 (v. 1.0) and monocle (v. 2.4) [25–28].

DEGs between the normoxia and hypoxia groups were used as ordering genes for subclustering in each cell cluster using monocle (v. 2.4). The tSNE and DDRTree algorithms were used to visualise data in each cluster. The subclusters were classified based on oxygen concentration, and states were classified based on subcluster and oxygen concentration. The progress of possible cell transitions was presented and inferred using the DDRTree graphs and states.

We ran velocyto.py (v. 11.2) annotator for each BAM file processed by CellRanger using the default parameters and a modified gencode vM12 gene transfer format file as described in a previous study [29]. The loom object files for each group were processed using velocyto.R (R package; v. 0.6). Monocle2 DDRTree representation was used to construct the final velocity plots embedded in cell-cell distance plots.

### 2.7. Functional Enrichment Analysis

As described in a previous research, the Kyoto Encyclopedia of Genes and Genomes (KEGG) and Gene Ontology (GO) pathway enrichment analyses were performed using the “clusterProfiler” R package [30–32]. The Benjamini–Hochberg method was employed to adjust the *p* values (adjust-*p* < 0.05 was considered significant). Gene set enrichment analysis (GSEA) was used to investigate potential mechanisms based on gene collection (c2.cp.v7.2.symbols.gmt [Curated]) from Molecular Signatures Database (MSigDB, https://www.gsea-msigdb.org/gsea/msigdb/index.jsp) using JAVA as described in previous studies [33, 34].

### 2.8. Statistical Analysis

The general information and other data of patients were used for statistical analyses using R version 4.0.2 (https://www.r-project.org/). For continuous variables, Student's *t*-tests were used to compare two distinct groups. For categorical variables, chi-square tests were performed. Logistic regression analysis was used to further screen the results of univariable analysis, and the R was used to establish a risk prediction model for the screened independent risk factors. In addition, the “rms” package and bootstrap internal validation method were used to verify the nomogram. The “ROCR” package was used to produce the ROC curves.

## 3. Results

### 3.1. Demographic Characteristics of GSE22255 and GSVA

Based on the inclusion and exclusion criteria, GSE22255 was, to the best of our knowledge, the only mRNA dataset that met our requirements. A total of 20 peripheral blood specimens from patients with IS and 20 specimens from a healthy control population were included. These 40 samples, 20 from men and 20 from women, were tested using the Affymetrix microarray platform. Details of the demographic characteristics are shown in Supplementary Table 1. A total of 186 KEGG pathway score matrices were obtained in the 40 samples using GSVA to score patients with IS. Various scores for each pathway in different subgroups suggested dissimilarities in the activity of each pathway between patients with IS and healthy subjects, which may be an important factor for determining the prognosis of patients after the onset of IS.

### 3.2. WGCNA Based on KEGG Pathway of GSE22255

The 186 pathways from 40 specimens obtained via GSVA were subjected to coexpression network construction. First, a soft threshold was calculated, and a scale-free topology model was built. The CC was greater than 0.85, and our data selection threshold was 9 (Supplementary Figure 1(a)). The pathways in the top 25% of variance were eventually filtered out, and a total of 18 pathway modules were selected according to their weight values (Supplementary Figure 1(b)). These modules were clustered, and the results are shown in Supplementary Figure 1(c). The tan module had the highest correlation with IS (CC = 0.39, *p* = 0.01; Supplementary Figure 1(d)) and a strong correlation with diabetes (CC = 0.32, *p* = 0.04). The two KEGG pathways included in the tan module were the calcium signaling and neuroactive ligand-receptor interaction pathways (Supplementary Figure 1(e)). We discovered that the calcium signaling pathway was correlated with both IS and diabetes, with a CC of 0.39 and a *p* value of <0.05, whereas the neuroactive ligand-receptor interaction pathway was correlated with only IS (CC = 0.37, *p* = 0.02).

### 3.3. Gene Modules Derived from WGCNA Based on Gene Expression of GSE22255

A coexpression network was constructed based on the gene expression matrix of the 40 specimens from GSE22255. Similar to the previous analysis, we calculated a soft threshold and built a scale-free topology model. The CC was greater than 0.85, and the data selection threshold was 6 (Supplementary Figure 2(a)). After weight-based filtering, we obtained a total of 14 modules (Supplementary Figure 2(b)), and the clustering of these modules is shown in Supplementary Figure 2(c). Furthermore, the correlation among the featured genes of each module is presented in Supplementary Figure 3(a). Eventually, the pink module was found to have the highest correlation with the KEGG calcium signaling pathway (CC = 0.33, *p* = 0.04; Supplementary Figure 2(d)). The following 15 genes were included in the pink module: *AC079305*.*10*, *ARMC8*, *ATF3*, *BCL10*, *BCL2A1*, *BRE*-*AS1*, *DYNLL2*, *EIF1*, *EREG*, *IL1B*, *MAP3K8*, *NFIL3*, *NR4A2*, *OTUD1*, and *PTGS2* (Supplementary Figure 2(e)). Furthermore, the calcium signaling pathway showed a significant positive correlation with *AC079305*.*10BCL10*, *BCL2A1*, *BRE*-*AS1*, *DYNLL2*, *EREG*, and *PTGS2* (*p* < 0.05).

### 3.4. GO and KEGG Enrichment Analyses

GO enrichment analysis of the calcium signaling pathway-related genes revealed that the genes were enriched in the apoptotic signaling pathway, cellular response to external stimuli, cytokine biosynthesis, cellular response to mechanical stimuli, fever generation, heat generation, and regulation of heat generation (*p* < 0.05; Supplementary Figure 3(b)). Furthermore, KEGG enrichment analysis revealed the following pathways (*p* < 0.05; Supplementary Figure 3(c)): NF-kappa B, C-type lectin, TNF, MAPK, IL-17, toll-like receptor, and T cell receptor signaling pathways and leishmaniasis. Therefore, calcium signaling pathway-related genes may be associated with the pathogenesis of IS by regulating these biological functions and pathways.

### 3.5. Characterisation of Hub Genes and miRNA-mRNA Network Construction

**Figure 1:**
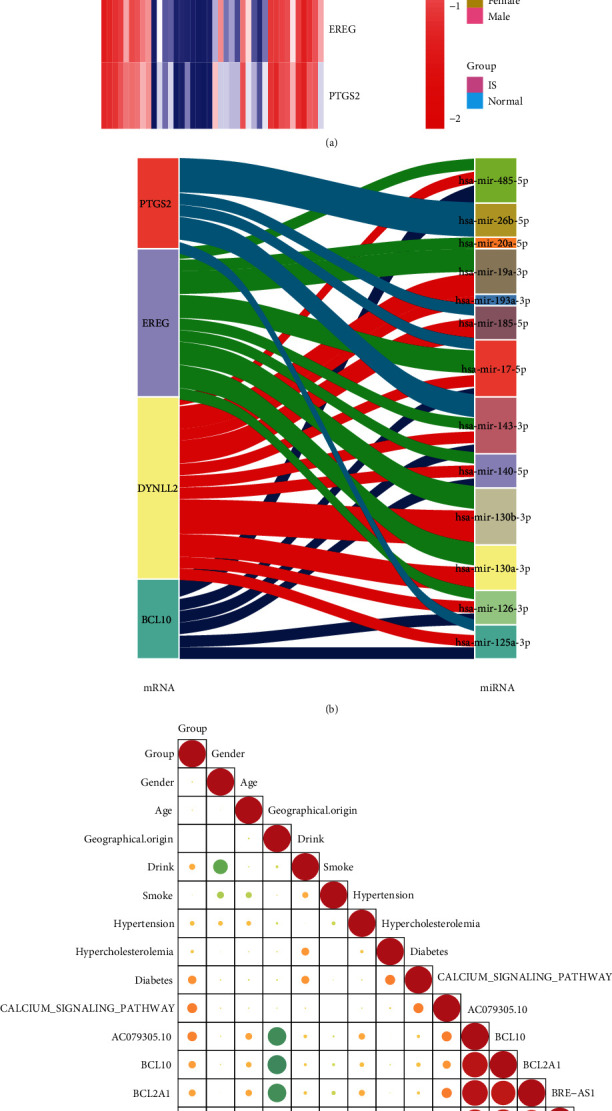
Characterisation of hub genes and miRNA-mRNA network construction. (a) A heat map of hub genes in the GSE22255 datasets. (b) A Sankey plot of miRNA-mRNA interaction for IS. (c) Correlation analysis of clinical features and hub genes. (d) Protein-protein interaction networks of hub genes.

The expression of hub genes in the GSE22255 dataset is shown in Figure 1(a). We obtained IS-associated miRNAs from the GSE110993 dataset. The miRWalk 3.0 and miRTarBase databases were used to construct the miRNA-mRNA network (Figure 1(b)). The correlation analysis of clinical features with central genes is shown in Figure 1(c). Consequently, we found a positive correlation among the expression of *AC079305.10*, *BCL10*, *BCL2A1*, *BRE-AS1*, *DYNLL2*, *EREG*, and *PTGS2*. In addition, protein-protein interaction (PPI) networks were mapped to describe the association among these hub genes (Figure 1(d)).

### 3.6. Construction of a Predictive Model of IS History

All features of the GSE22255 dataset were used to perform a multivariable logistic analysis to plot a nomogram-based prediction model (Supplementary Figure 4(a)) (Supplementary Tables 2 and 3). The ROC curve is shown in Supplementary Figure 4(b) (AUC = 0.8325). The calibration curve suggested that the model had good predictive ability (Supplementary Figure 4(c)). In addition, the C-index of the model was 0.832 (95% CI, 0.704–0.960), which represented a good predictive performance. The decision curve reflected that the model had good clinical applicability (Supplementary Figure 4(d)).

### 3.7. Single-Cell Analysis

**Figure 2:**
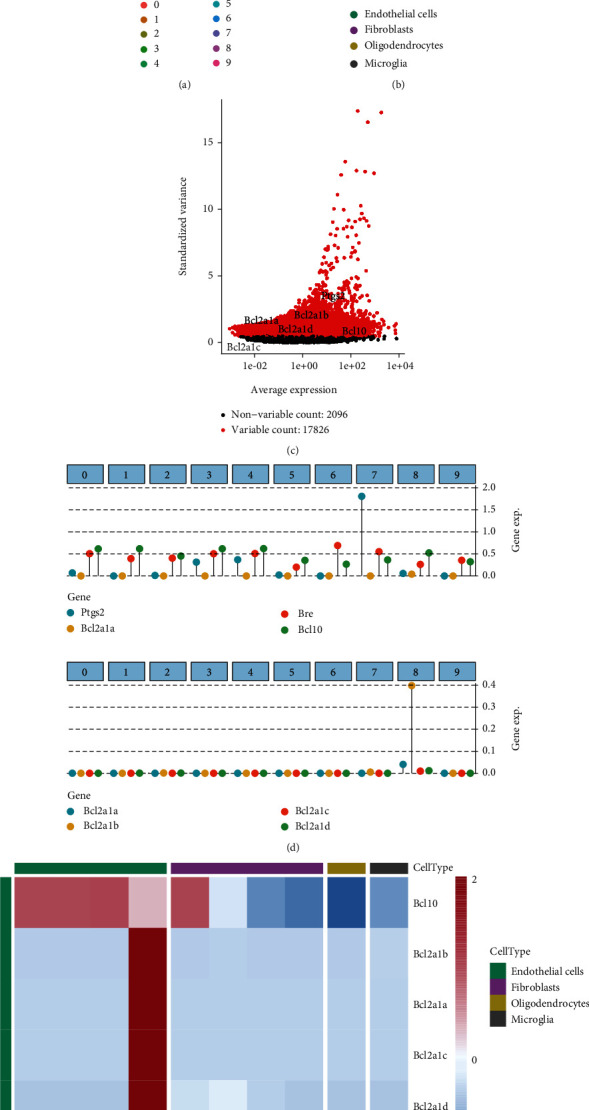
Expression of hub genes in the mouse brain vasculature. (a, b) UMAP analysis showing the results of descending clustering and annotation of cell subpopulations (endothelial cells, fibroblasts, oligodendrocytes, and microglia) in cells of the mouse cerebrovascular system. (c) ANOVA analysis showing the variation of gene expression in the mouse cerebrovascular system cells. Variable genes are indicated by red dots according to selected thresholds, and nonvariable genes are indicated by black dots. The relevant hub genes (*Bcl10*, *Bcl2A1a*, *Bcl2A1b*, *Bcl2A1c*, *Bcl2A1d*, *Bre*, *Dynll2*, and *Ptgs2*) are indicated in the figure. (d) Relative expression of related hub genes in various cell subpopulations. (e) A heat map showing the relative expression of each molecule in cell subpopulations and cell clusters. The relative expression profiles of marker genes associated with each cell subpopulation are known.

**Figure 3:**
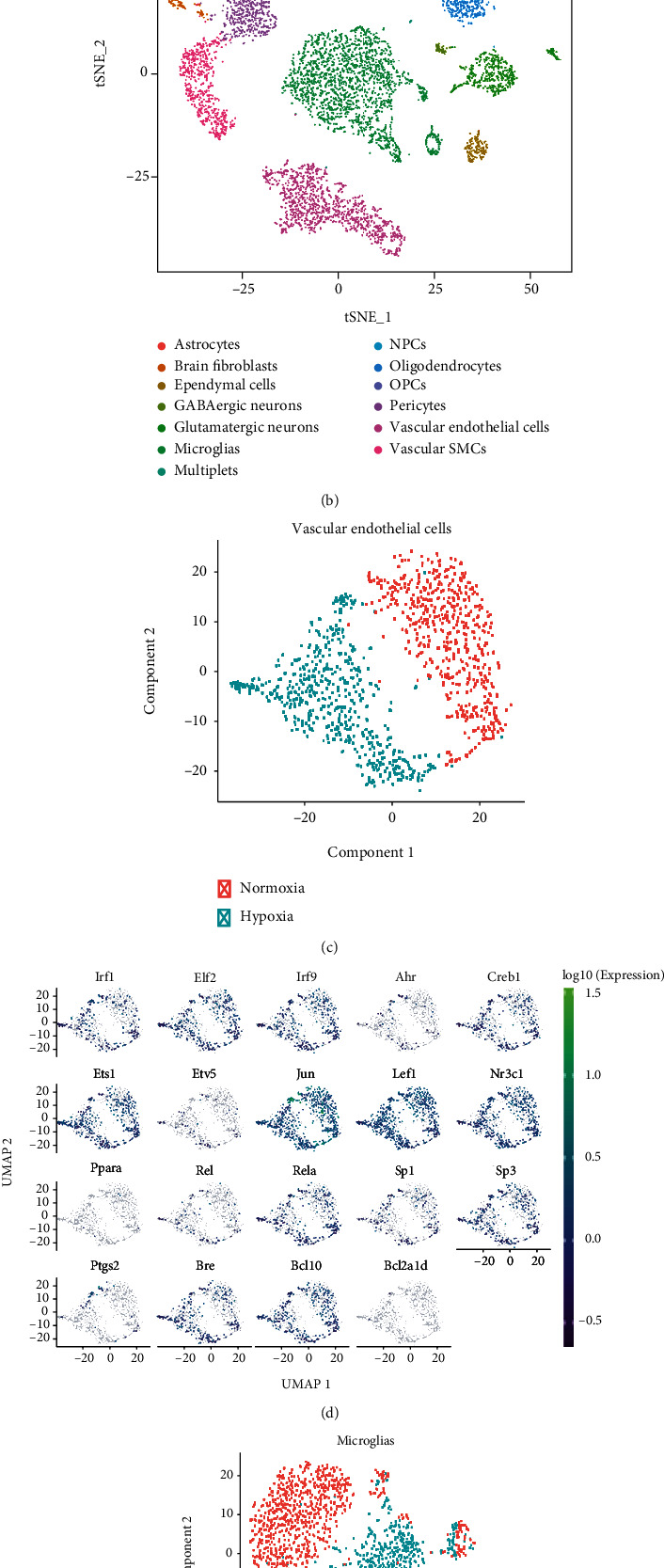
Pseudotemporal analysis in the mouse cerebral cortex. (a, b) Visualisation of clustering and annotation in the tSNE plot. (c) UMAP plot of vascular endothelial cells in hypoxia and normoxia. (d) Expression of 19 hub TFs and targeted genes in vascular endothelial cells. (e, f) Same as (c–d) but for microglia.

The results of single-cell downscaling and clustering revealed 10 cell clusters and 4 cell subgroups in GSE98816 (Figures 2(a) and 2(b)). Annotation of cell clusters using SingleR is shown in Supplementary Figure 5(a).  Figure 2(c) shows the differential expression of relevant core genes in the cells. We further analysed the differential expression of related genes in cell subpopulations (Figures 2(d) and 2(e)) and found that *Ptgs2* was specifically expressed in microglia. In addition, the *Bcl* family genes (*Bcl10*, *Bcl2A1a*, *Bcl2A1b*, *Bcl2A1c*, and *Bcl2A1d*) were mainly expressed in endothelial cells, and *Bre* was mainly expressed in oligodendrocytes. These results revealed that the key genes in our screened pathways were specifically expressed at the single-cell level, thus providing a direction for subsequent in-depth studies targeting specific cells. The processes of dimensional reduction and cluster annotation of scRNA-seq data in GSE125708 are shown in Supplementary Figures 6–7, and the cell clustering and annotation results revealed a total of 18 cell clusters, and 13 annotated cell subpopulations were obtained (Figures 3(a) and 3(b)).

### 3.8. Identification of TF-Target Gene Pairs Using SCENIC

**Figure 4:**
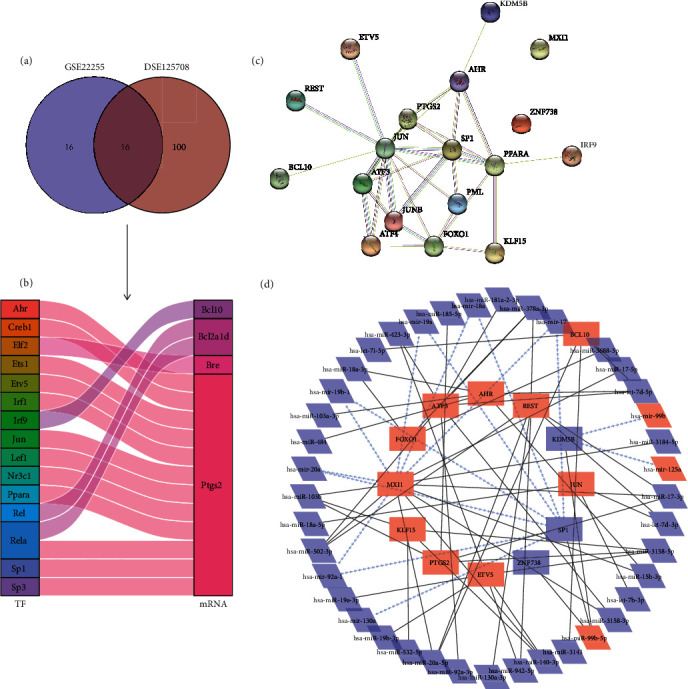
Analysis of the TF-mRNA and miRNA-TF networks. (a) A Venn plot of TF-target mRNA intersection analysis based on data from the GSE22255 and GSE125708 datasets using SCENIC. (b) A Sankey plot showing 16 TF-mRNA pairs. DEGs between patients with IS and healthy controls are noted in bolded italics. (c) Protein-protein interaction (PPI) networks of the 16 TF-mRNA pairs. (d) PPI networks of TFs and miRNAs. The black solid line represents miRNA-TF regulatory pairs, while the blue dashed arrows represent TF-miRNA regulatory relationships.

The SCENIC analysis of GSE98816 and GSE125708 revealed 32 and 116 TF-targeted gene pairs, respectively (Figure 4(a)). Among these gene pairs, 16 hub TF-target gene pairs were shared by both datasets (Figure 4(b)). In these 16 gene pairs, the TFs *AHR*, *ETV5*, *IRF9*, *JUN*, *PPARA*, and *SP1* and the targeted genes *BCL10* and *PTGS2* differed between patients with IS and healthy subjects in GSE22255. Figure 4(c) demonstrates a PPI network constructed based on these 16 TF-target gene pairs, with *SP1*, *JUN*, *PPARA*, and *PTGS2* at the centre of the network. The key TFs and corresponding target genes and differentially expressed miRNAs were used for constructing an interaction network (Figure 4(d)).

### 3.9. Regulons in the Mouse Brain Vasculature

**Figure 5:**
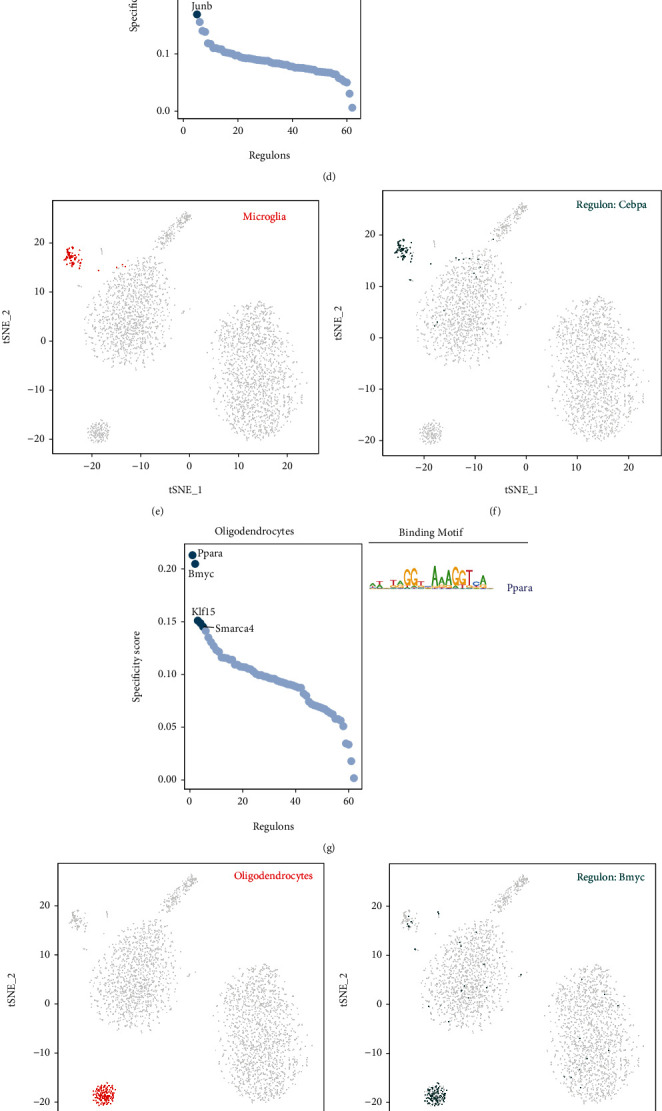
Cell type-specific regulons in the mouse brain vasculature. (a) Ranks of regulons in the mouse brain vasculature endothelial cells sorted based on regulon specificity scores (right) and the corresponding binding motifs of TFs. (b) Endothelial cells are highlighted as red dots in the tSNE plot. (c) The expression values of interesting genes are presented as green dots in the tSNE plot. (d–f) Same as (a–c) but for microglia. (g–i) Same as (a–c) but for oligodendrocytes. (j–l) Same as (a–c) but for fibroblasts.

Based on the RSS scores, mouse cerebrovascular cells were clustered (Supplementary Figure 5(b)), and M1–M6 modules were constructed (Supplementary Figure 5(c)). The heat map of scores for these modules is shown in Supplementary Figure 5(d). Figures 5(a)–5(c) reveal that the top five TFs with major transcriptional regulatory roles in endothelial cells are *Lef1*, *Irf7*, *Fli1*, *Stat1*, and *Elk3*. In microglia, *Cebpa*, *Spi1*, *Rest*, *Maf*, and *Junb* were the major regulatory TFs (Figures 5(d)–5(f)). The most relevant specific regulators of oligodendrocytes were *Ppara*, *Bmyc*, *Klf15*, *Smarca4*, and *Maf* (Figures 5(g)–5(i)). In fibroblasts, *Jund*, *Sp1*, *Klf15*, *Bclaf1*, and *Atf1* were identified as important regulatory TFs (Figures 5(j)–5(l)).

### 3.10. Regulons in the Mouse Cerebral Cortex

**Figure 6:**
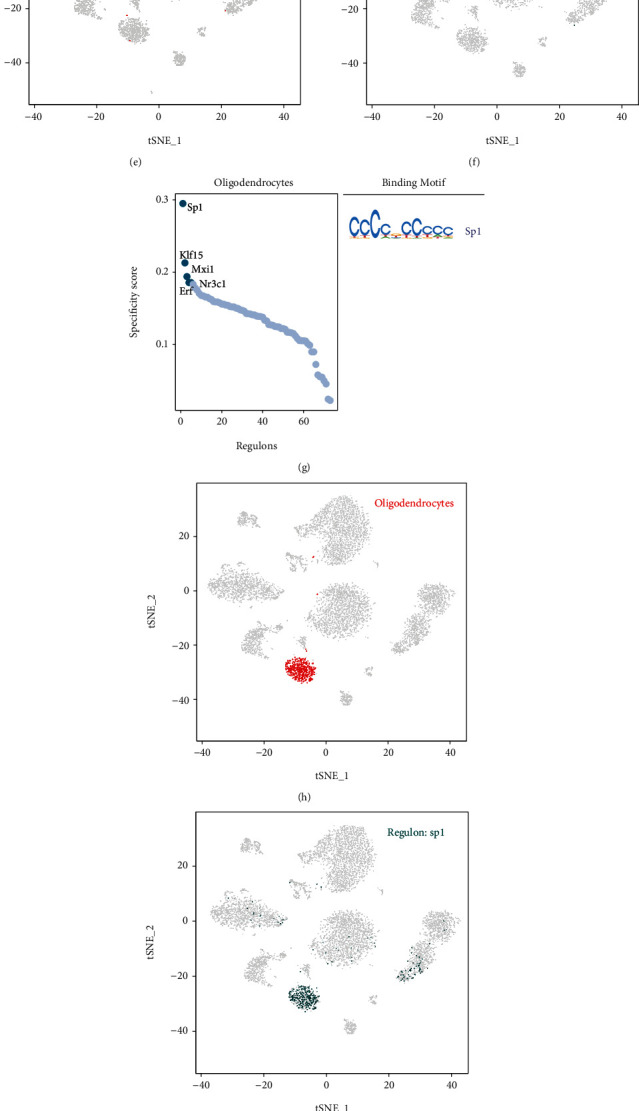
Cell type-specific regulons in the mouse cerebral cortex. (a) Ranks of regulons in the mouse cerebral cortex vascular endothelial cells sorted based on regulon specificity scores (right), and the corresponding binding motifs of TFs. (b) Vascular endothelial cells are highlighted as red dots in the tSNE plot. (c) The expression values of interesting genes are presented as green dots in the tSNE plot. (d–f) Same as (a–c) but for microglia. (g–i) Same as (a–c) but for oligodendrocytes. (j–l) Same as (a–c) but for astrocytes.

Figures 6(a)–6(c) demonstrate the top five TFs, namely, *Lef1*, *Ppard*, *Elk3*, *Irf9*, and *Ets1*, with major transcriptional regulatory roles in vascular endothelial cells. In microglia, *Etv3*, *Maf*, *Irf5*, *Ikzf1*, and *Runx1* were the major regulatory TFs (Figures 6(d)–6(f)). The most relevant specific regulators of oligodendrocytes were *Sp1*, *Klf15*, *Mxi1*, *Erf*, and *Nr3c1* (Figures 6(g)–6(i)). In astrocytes, *Klf15*, *Foxo1*, *Bmyc*, *Zfp595*, and *Kdm5a* were identified as important regulatory TFs (Figures 6(j)–6(l)). In vascular SMCs, *Nfatc4*, *Ppara*, *Bcl3*, *Pml*, and *Atf3* were identified as the most important regulatory TFs. In pericytes, *Ppara*, *Nfatc4*, *Pml*, *Bcl3*, and *Ets1* were identified as important TFs. *Ppara* expression was found to be significantly specific in both vascular endothelial cells and pericytes. In glutamatergic neurons, *Egr4*, *Etv5*, *Kdm5b*, *Ahr*, and *Bmyc* were among the important TFs. In fibroblasts, *Nfatc4*, *Zfp595*, *Zfp110*, *Sp3*, and *Bcl3* were identified as important TFs of key genes (Supplementary Figure 8). These results suggest that GRNs are different in different cell types.

### 3.11. Pseudotemporal Trajectory Inference Analysis in the Mouse Brain Vasculature

**Figure 7:**
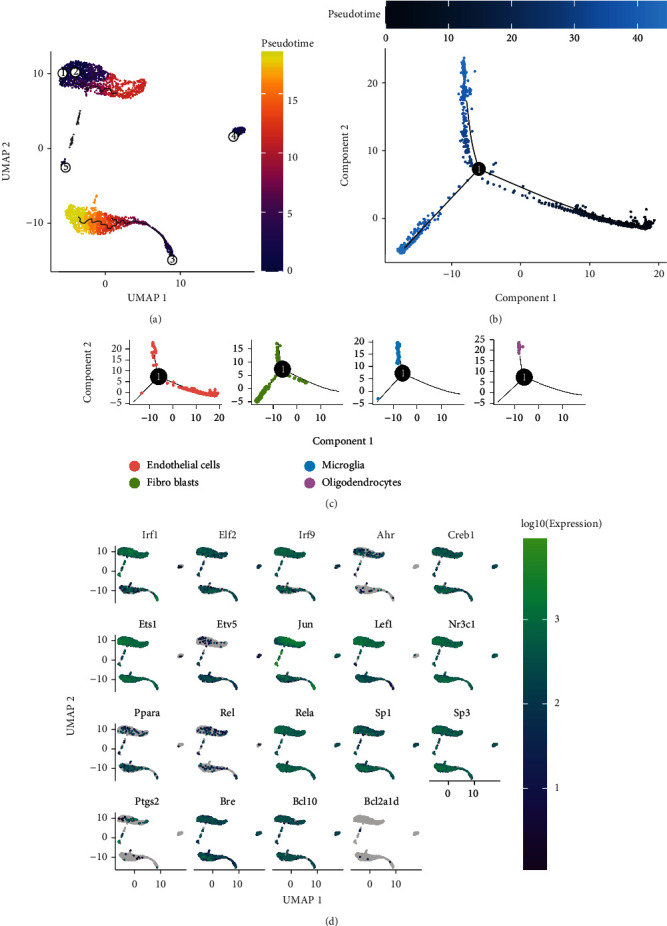
Pseudotemporal trajectory inference analysis in the mouse brain vasculature. (a) Trajectory inference in each of the four cell types. (b) Pseudotime of cells in the DDRTree plot. (c) State distribution of each cell type in the DDRTree plot. (d) Expression of hub TFs and targeted genes in mouse brain vasculature cells.

The trajectories of each cell population are shown in Figure 7(a).  Figure 7(b) shows the pseudotime of cells in the DDRTree plot, and Figure 7(c) shows the states of each cell type. Endothelial cells and fibroblasts had their respective developmental trajectories in addition to overlapped trajectories with microglia and oligodendrocytes.  Figure 7(d) demonstrates the expression of the 16 hub TFs and targeted genes.

### 3.12. Pseudotemporal Analysis of Mouse Cerebral Cortex Cells in Hypoxia and Normoxia

**Figure 8:**
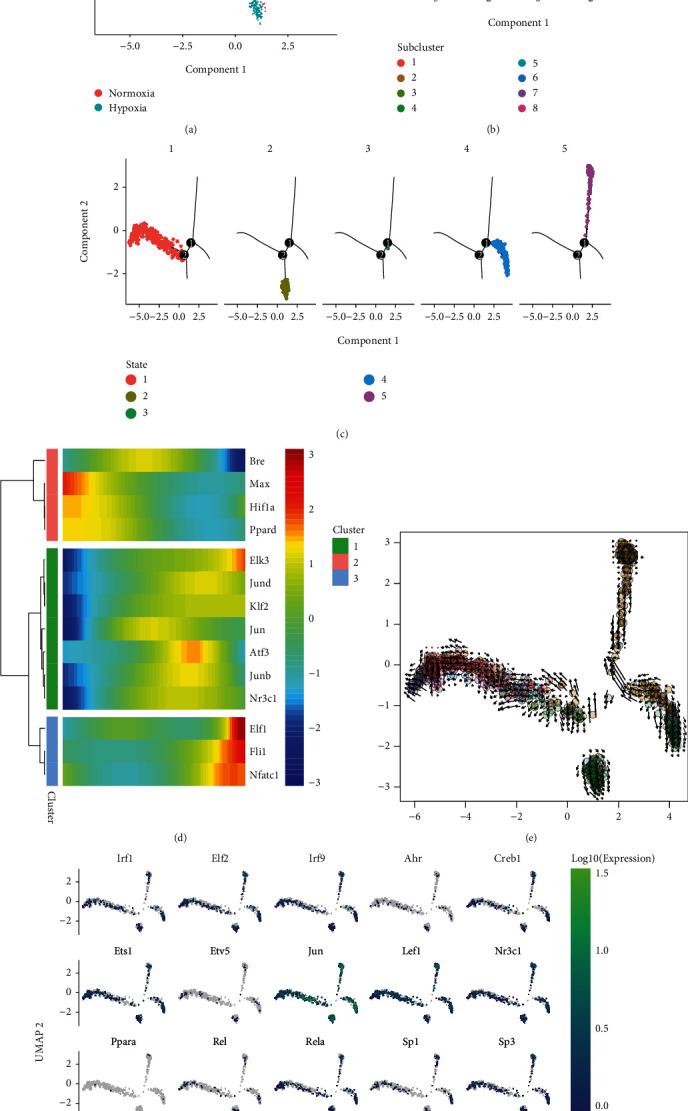
Pseudotemporal and RNA velocity analyses of vascular endothelial cells. (a) DDRTree plot of endothelial cells in normoxia and hypoxia. (b) Visualisation of subcluster analysis in the DDRTree plot. (c) State distribution of vascular endothelial cells in the DDRTree plot. (d) A heat map showing pseudotime-related genes among the 19 hub TFs and target genes. (e) RNA velocity plot with longer arrows representing stronger transcriptional activity. (f) Expression of the 19 hub TFs and target genes in vascular endothelial cells.

We further investigated the changes in gene expression at the single-cell level in the cerebral cortex under hypoxic and normoxic conditions. UMAP analysis revealed evident differences in vascular endothelial cells between the normoxia and hypoxia groups (Figure 3(c)), and the corresponding expression of hub genes between the two groups is shown in Figure 3(d). Figures 3(e) and 3(f) demonstrate the results of UMAP analysis of microglia. The vascular endothelial cells were well separated between the normoxia and hypoxia groups based on pseudotime (Figures 8(a) and 8(b)).  Figure 8(c) shows the states of vascular endothelial cells under hypoxic and normoxic conditions. *Jun* and *Nr3c1* were found to play an important role in cellular transformation from hypoxia to normoxia (Figure 8(d) and Supplementary Figure 9). RNA velocity analysis of the two groups is shown in Figure 8(e). Different RNA velocity was found in different subclusters, which revealed transcriptional heterogeneity in vascular endothelial cells. The expression of hub TFs and target genes is shown in Figure 8(f).

**Figure 9:**
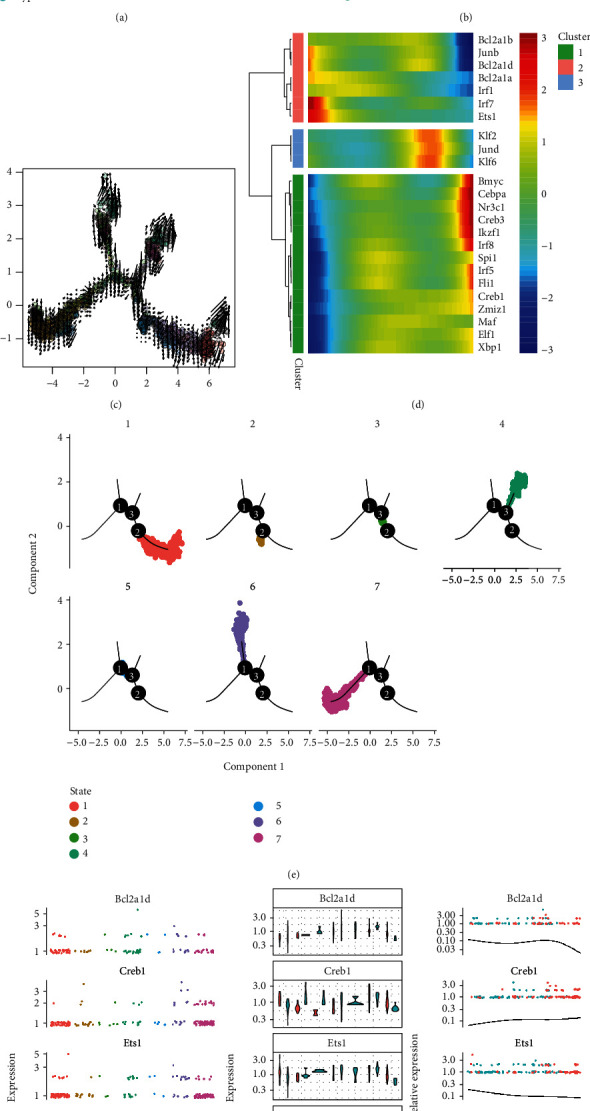
Pseudotemporal and RNA velocity analyses of microglia. (a) DDRTree plot of microglia in normoxia and hypoxia. (b) Visualisation of subcluster analysis in the DDRTree plot. (c) RNA velocity plot with longer arrows representing stronger transcriptional activity. (d) A heat map showing pseudotime-related genes among the 19 hub TFs and target genes. (e) State distribution of microglia in the DDRTree plot. (f) Expression of the 19 hub TFs and targeted genes in microglia.

Furthermore, the same analysis was performed in microglia (Figures 9(a) and 9(b)). Evident heterogeneous differentiation of microglia was observed in state 7 between the normoxia and hypoxia groups, whereas a transition from hypoxia to normoxia was observed in state 1 (Figures 9(c) and 9(e)). In addition, cells in the hypoxic state had increased RNA velocity than that in the normoxic state, suggesting stronger transcriptional activity and a high number of unspliced RNAs. This finding suggests that hypoxia has a negative effect on the transformation of microglia from hypoxia to normoxia. *Bcl2a1d*, *Creb1*, *Ets1*, *Irf1*, and *Nr3c1* were identified as pseudotemporal-related markers in microglia during the transformation (Figure 9(f)). Figures 6(d) and 9(d) demonstrate that *Maf*, *Irf5*, and *Ikzf1* play an important role in cell state transition between hypoxia and normoxia.

A similar analysis was also performed for astrocytes and pericytes (Supplementary Figures 10, 11, and 12). Astrocytes showed distinctly different differentiation trajectories and RNA velocity in hypoxia and normoxia (Supplementary Figures 10(e) and 10(f)). *Bmyc*, *Ep300*, *Etv5*, *Irf9*, *Jun*, *Nr3c1*, and *Uqcrb* were the intersecting genes of key genes and pseudotemporal-related genes (Supplementary Figure 11(e)). Furthermore, pericytes also had different differentiation trajectories in normoxia and hypoxia (Supplementary Figures 12 (a–c)). *Hif1a*, *Klf6*, *Nr3c1*, *Smarca4*, and *Uqcrb* were identified as important pseudotime-related genes between the two groups. Moreover, the pseudotime of pericytes was longer in hypoxia than in normoxia. This finding suggests that pericytes play a role in responses to hypoxia (Supplementary Figure 12(e)).

### 3.13. Differentially Expressed Hub Genes in Clinical Cohorts

**Figure 10:**
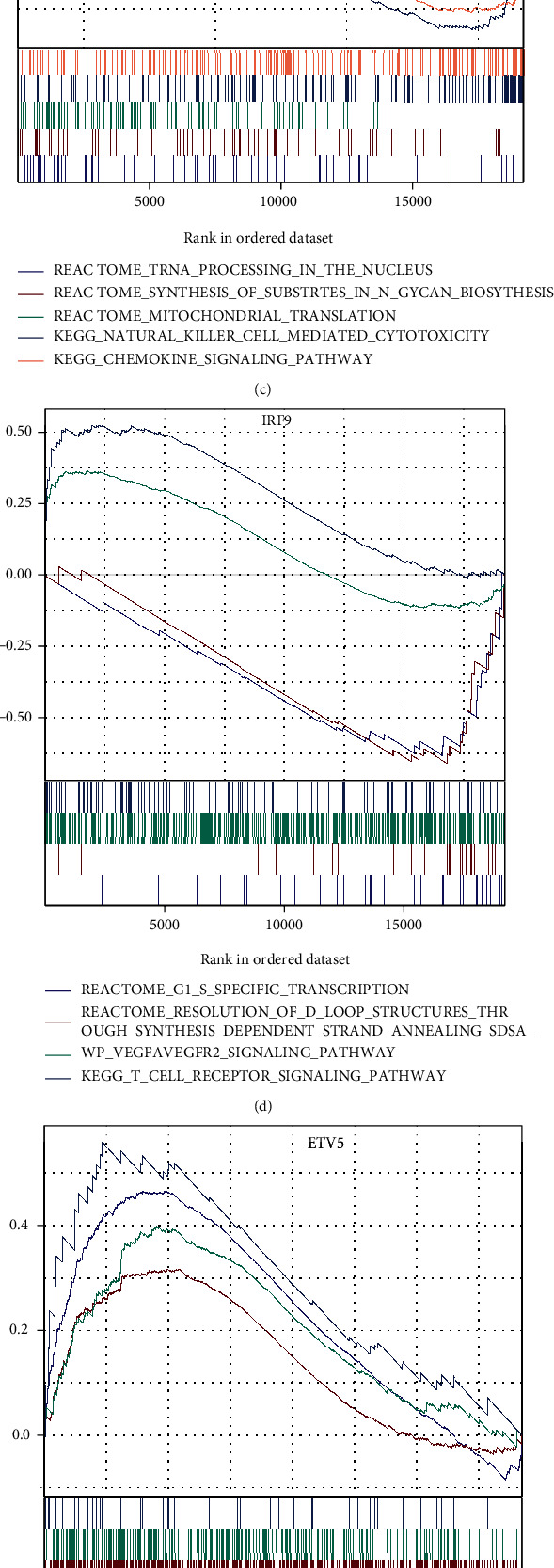
Differential analysis of hub TFs and GSEA in clinical cohorts. (a) A heat map showing the expression of differentially expressed hub TFs between patients with IS and healthy controls. (b) A volcano plot showing the distribution in fold change of hub TFs. (c–f) GSEA of *JUN*, *IRF9*, *ETV5*, and *PPARA*. Pathways with upward curves are enriched in samples with downregulated gene expression.

The heat map of differentially expressed hub genes in clinical samples is demonstrated in Figure 10(a), whereas the differential hub TFs are presented in Figure 10(b). Combined with the previous pseudotemporal trajectory and SCENIC analyses, *JUN*, *IRF9*, *ETV5*, and *PPARA* were subjected to GSEA. The GSEA of *JUN* revealed that tRNA processing in the nucleus, synthesis of substrates in N-glycan biosynthesis, and mitochondrial translation were downregulated in IS, whereas natural killer cell-mediated cytotoxicity and chemokine signaling pathway were upregulated. The GSEA of *IRF9* revealed that G1/S-specific transcription and resolution of D-loop structures through synthesis-dependent strand annealing (SDSA) were downregulated in IS, whereas the VEGFA-VEGFR2 signaling pathway and T cell receptor signaling pathway were upregulated (FDR < 0.05). The enrichment pathways of *ETV5* and *PPARA* are shown in Figures 10(e) and 10(f) (*p* < 0.05).

## 4. Discussion

In this study, WGCNA of 186 pathways in 40 specimens obtained via GSVA of GSE22255 yielded 18 pathway modules; of which, the tan module was most significantly positively correlated with IS. The two KEGG pathways, calcium signaling and neuroactive ligand-receptor interaction pathways, were included in the tan module. The calcium signaling pathway was found to be significantly positively correlated with the pink module in the subsequent WGCNA based on the gene expression of GSE22255. In addition, the pink module had the highest correlation with IS. Furthermore, we found that the calcium signaling pathway was positively correlated with genes in the pink module, including *AC079305*.*10*, *BCL10*, *BCL2A1*, *BRE*-*AS1*, *DYNLL2*, *EREG*, and *PTGS2*. An IS-associated miRNA-mRNA regulatory network was also constructed, which revealed the core pathways and key genes that may be altered after the onset of IS. In addition, we used SCENIC to identify key TFs regulating these genes using mouse scRNA-seq data, followed by pseudotemporal trajectory inference analysis to assess the role of these TFs and target genes at the single-cell level under normoxic and hypoxic conditions.

We established that the calcium signaling pathway activity was associated with the development of IS. Calcium ion overload leads to cell necrosis or apoptosis [35]. Furthermore, the upregulation of calcium ions in the cell plasma increases, leading to platelet activation and thrombosis, suggesting that overactivation of the calcium signaling pathway is associated with both thrombosis and platelet activation [36]. However, a deficiency of calcium channels may exhibit neurovascular protective activity [37]. Recent meta-analyses have confirmed that some calcium channels exert a vascular protective effect against stroke. In conclusion, our findings are consistent with those of related results, revealing that the calcium signaling pathway is a risk factor associated with IS and may be associated with poor prognosis after the development of IS in patients.

Calcium signaling pathway-related genes (*AC079305.10*, *BCL10*, *BCL2A1*, *BRE-AS1*, *DYNLL2*, *EREG*, and *PTGS2*) are related to apoptosis after stroke, whereas posttranscriptional regulation of *BCL2A1* may be possibly associated with IS [38, 39]. It has been proposed that *EREG* genes may be associated with homeostatic imbalances in immune and inflammatory function after IS, and the *PTGS2* gene may be a risk factor for the development of IS [40–42]. In this study, calcium signaling pathway-related genes (*AC079305.10*, *BCL10*, *BCL2A1*, *BRE-AS1*, *DYNLL2*, *EREG*, and *PTGS2*) were found to be significantly enriched in the NF-kappa B, C-type lectin receptor, TNF, and MAPK signaling pathways. Existing studies indicate that activation of the NF-kappa B/MAPK signaling pathway promotes the activation of inflammatory vesicles in neurons in the brain, exacerbating the neurological damage caused by IS [43]. In addition, studies have shown that sevoflurane inhibits the NF-kappa B/MAPK signaling pathway, which in turn significantly alleviates cerebral oedema and cerebral infarction after IS. Sevoflurane also moderates the death of astrocytes, neurons, and vascular endothelial cells, thereby playing a neuroprotective role in IS [44]. Therefore, inhibition of the NF-kappa B/MAPK signaling pathway can reduce IS-induced brain damage. Numerous studies have confirmed that overactivation of these pathways is associated with inflammatory responses and further thrombogenesis [45–48]. Recent studies have reported that proinflammatory signals from immune cells after the onset of IS further exacerbate brain injury. Understanding the changes in inflammatory mediators after the onset of IS may help to develop inflammation-related neuroprotective strategies for poststroke complications [49]. In this study, the GO enrichment analysis revealed that calcium signaling pathway-related genes might be involved in apoptosis-related processes, suggesting that the occurrence of IS is closely related to apoptosis. Increased levels of apoptosis are one of the serious consequences of IS [50].

We further analysed the TFs of hub genes at the single-cell level. Vascular endothelial cells perform several functions, including regulating vascular wall contraction based on chemical, physical, or electrical signals; participating in inflammatory responses; stimulating neoangiogenesis; and regulating vascular barrier permeability [51]. Hypoxia is an important stimulus for promoting vascular neovascularization and also alters endothelial cell function, metabolism, and migration [52, 53]. Our study suggested that *Jun* and *Junb* were pseudotime-associated genes in mouse endothelial cells under normoxic and hypoxic conditions. Previous studies have reported that the JNK/c-Jun/AP-1 pathway can be regulated via IL-13 in human vascular endothelial cells to participate in vascular inflammation-related pathophysiological alterations [54]. *JUNB* plays an important role in the production of vascular endothelial growth factor (VEGF), which induces *JUNB* expression in the budding vascular adventitia [55–57]. During mouse development, *Junb* expression is elevated in vascular endothelial cells owing to neurovascular interactions and is involved in the embryonic vascular network construction [58]. In our study, *Jun* and *Junb* expressions were downregulated in vascular endothelial cells in hypoxia. In addition, *Junb*-deficient vascular SMCs and endothelial cells were found to have impaired motility owing to the failure of stress fibre formation [59]. In microglia, ATP can contribute to the early transcriptional accumulation of *JUNB*, thereby activating microglia [60]. The GSEA of *JUN* suggested a disturbance of cellular energetic balance in IS. The association between JUN and cellular energetic balance needs further investigation, including proteins involved in oxidative phosphorylation. In addition, REST was found to be a possible inhibitor of microglial migration [61].

Astrocytes perform diverse functions, which can be both beneficial and harmful [62]. *Jun*, *Irf9*, and *Etv5* are putative time-related TFs grouped according to oxygen concentration. Astrocytes can induce proliferation and reduce cellular autophagy by activating c-Jun N-terminal kinase [63]. To the best of our knowledge, this is the first study to report that *IRF9* and *ETV5* might be astrocyte-specific TFs associated with oxygen concentration in IS. The GSEA of *IRF9* revealed that G1/S-specific transcription and resolution of D-loop structures through synthesis-dependent strand annealing (SDSA) were downregulated, whereas the VEGFA-VEGFR2 signaling pathway was upregulated in IS. *Etv5*/*Erm* promotes astrocyte production and is regulated by *MEK* [64]. During the differentiation of neural progenitor cells (NPCs) into neurons, *ETV5* prevents the production of glutamatergic neurons and increases the number of GABAergic subtype neurons from NPCs [65]. The GSEA of *ETV5* revealed that neutrophil degranulation and amino sugar and nucleotide sugar metabolism were downregulated in IS. *Sp1* is an important transcriptional regulator of oligodendrocytes and fibroblasts. Sp1 phosphorylation can regulate oligodendrocyte differentiation by regulating *MBP* transcription and is also an important factor in the regulatory network mediating the differentiation of NSCs [66, 67]. The GSEA of *PPARA* revealed that cytokines and inflammatory response were upregulated in IS.

Numerous factors are now known to be associated with IS [68–70]. In recent years, several studies have identified genes that may be associated with the development and prognosis of IS [71–74]. These studies have played a key role in the clinical treatment of IS. Concerting with the calcium signaling pathway, *BCL10*, *BCL2A1*, and *EREG* were used to build a predictive model for IS history. Furthermore, miRNAs have also been found to play a crucial role in the nervous system. Therefore, an IS-associated miRNA-mRNA interaction network was constructed in this study [75]. Possibly a post-IS regulatory network may also be involved.

We first used GSVA to reveal the underlying mechanisms of IS associated with the KEGG pathway, providing a guiding study for further research. In this study, we identified new key genes and signaling pathways by integrating WGCNA, suggesting that bioinformatics-based WGCNA is an excellent approach to reveal the molecular mechanisms of IS-related disorders. To the best of our knowledge, we have reported for the first time that the calcium signaling pathway and the genes of the pink module (including *AC079305.10*, *BCL10*, *BCL2A1*, *BRE-AS1*, *DYNLL2*, *EREG*, and *PTGS2*) were significantly correlated with IS. Enrichment analyses also suggested that alterations in the microenvironment after the onset of IS further increased the risk of thrombosis, which may be related to the inflammatory responses. Finally, we analysed the role of TFs of these core genes at the single-cell level using SCENIC and pseudotemporal trajectory inference analyses. *Jun* and *Junb* were identified to be associated with hypoxia in endothelial cells, and *Irf9* and *Etv5* were identified as astrocyte-specific TFs associated with oxygen concentration. These results offer clues to understand IS as well as its treatment and prognosis.

However, the findings of this study require to be validated in studies with large sample size, which was limited in this study by a restricted database. In addition, the pathways and genes that were screened require further confirmation. In future studies, we will continue to collect blood samples and clinical information from patients with IS to further validate and optimise the foundation of this study. To the best of our knowledge, for the first time, we have identified a correlation between the calcium signaling pathway and the genes of the pink module; however, this correlation requires validation by cellular and animal experiments. We further assessed the expression of TFs that regulate these genes at the single-cell level. In conclusion, further studies are warranted to assess the specific regulatory mechanisms of these genes and pathways.

## 5. Conclusion

The main objective of this study was to identify characteristic alterations in the post-IS microenvironment. Genes of the calcium signaling pathway and pink module (including *AC079305.10*, *BCL10*, *BCL2A1*, *BRE-AS1*, *DYNLL2*, *EREG*, and *PTGS2*) and related TFs (*JUN*, *IRF9*, *ETV5*, and *PPARA*) were identified as possible core pathways and genes of the altered blood microenvironment after IS. *Jun* and *Junb* were identified to be associated with hypoxia in endothelial cells, and *Irf9* and *Etv5* were identified as astrocyte-specific TFs associated with oxygen concentration in the mouse cerebral cortex. This study provides clues to understanding the pathogenesis of IS and developing new diagnostic and therapeutic strategies.

## Data Availability

All data used in this paper are from the GSE22255, GSE110993, GSE98816, and GSE125708 datasets of the GEO database.

## Abbreviations

GEO: Gene Expression Omnibus
GO: Gene Ontology
GSVA: Gene set variation analysis
GSEA: Gene set enrichment analysis
IS: Ischemic stroke
KEGG: Kyoto Encyclopedia of Genes and Genomes
WGCNA: Weighted gene coexpression network analysis
CC: Correlation coefficient
scRNA-seq: Single-cell RNA sequencing
SCENIC: Single-cell regulatory network inference and clustering
TFs: Transcription factors.

## Supplementary Materials

Supplementary Materials Supplementary Figure 1: pathway modules derived from WGCNA based on the KEGG pathway analysis of GSE22255. Supplementary Figure 2: gene modules based on the gene expression of GSE22255. Supplementary Figure 3: relationship among gene module eigengenes and enrichment analyses of the calcium signaling pathway-related genes. Supplementary Figure 4: constructing a predictive model of IS history. Supplementary Figure 5: identification of regulon modules based on the regulation CSI matrix in mouse brain vasculature cells. Supplementary Figure 6: integration, dimensionality reduction, and cluster annotation of scRNA-seq data in the mouse cerebral cortex. Supplementary Figure 7: annotation of scRNA-seq data in the mouse cerebral cortex using scmap. Supplementary Figure 8: cell type-specific regulons in the mouse cerebral cortex. Supplementary Figure 9: differential expression of pseudotime-related molecules in the states and between the two groups. Supplementary Figure 10: UMAP plot of mouse cerebral cortex cells. Supplementary Figure 11: pseudotemporal and RNA velocity analyses of astrocytes. Supplementary Figure 12: pseudotemporal and RNA velocity analyses of pericytes. Supplementary Table 1: demographic characteristics of GSE22255. Supplementary Table 2: univariable logistic regression analysis. Supplementary Table 3: multivariable logistic regression analysis.
