# Supplementary material for: Role of Calcium Signaling Pathway-Related Gene Regulatory Networks in Ischemic Stroke Based on Multiple WGCNA and Single-Cell Analysis
Source: Oxid Med Cell Longev. 2021 Dec 26;2021:8060477. doi: 10.1155/2021/8060477 (PMC8720592; doi:10.1155/2021/8060477)
Supplement: Supplementary Materials — Supplementary Figure 1: pathway modules derived from WGCNA based on the KEGG pathway analysis of GSE22255. Supplementary Figure 2: gene modules based on the gene expression of GSE22255. Supplementary Figure 3: relationship among gene module eigengenes and enrichment analyses of the calcium signaling pathway-related genes. Supplementary Figure 4: constructing a predictive model of IS history. Supplementary Figure 5: identification of regulon modules based on the regulation CSI matrix in mouse brain vasculature cells. Supplementary Figure 6: integration, dimensionality reduction, and cluster annotation of scRNA-seq data in the mouse cerebral cortex. Supplementary Figure 7: annotation of scRNA-seq data in the mouse cerebral cortex using scmap. Supplementary Figure 8: cell type-specific regulons in the mouse cerebral cortex. Supplementary Figure 9: differential expression of pseudotime-related molecules in the states and between the two groups. Supplementary Figure 10: UMAP plot of mouse cerebral cortex cells. Supplementary Figure 11: pseudotemporal and RNA velocity analyses of astrocytes. Supplementary Figure 12: pseudotemporal and RNA velocity analyses of pericytes. Supplementary Table 1: demographic characteristics of GSE22255. Supplementary Table 2: univariable logistic regression analysis. Supplementary Table 3: multivariable logistic regression analysis. [file 8060477.f1.docx]

# Supplementary Figures


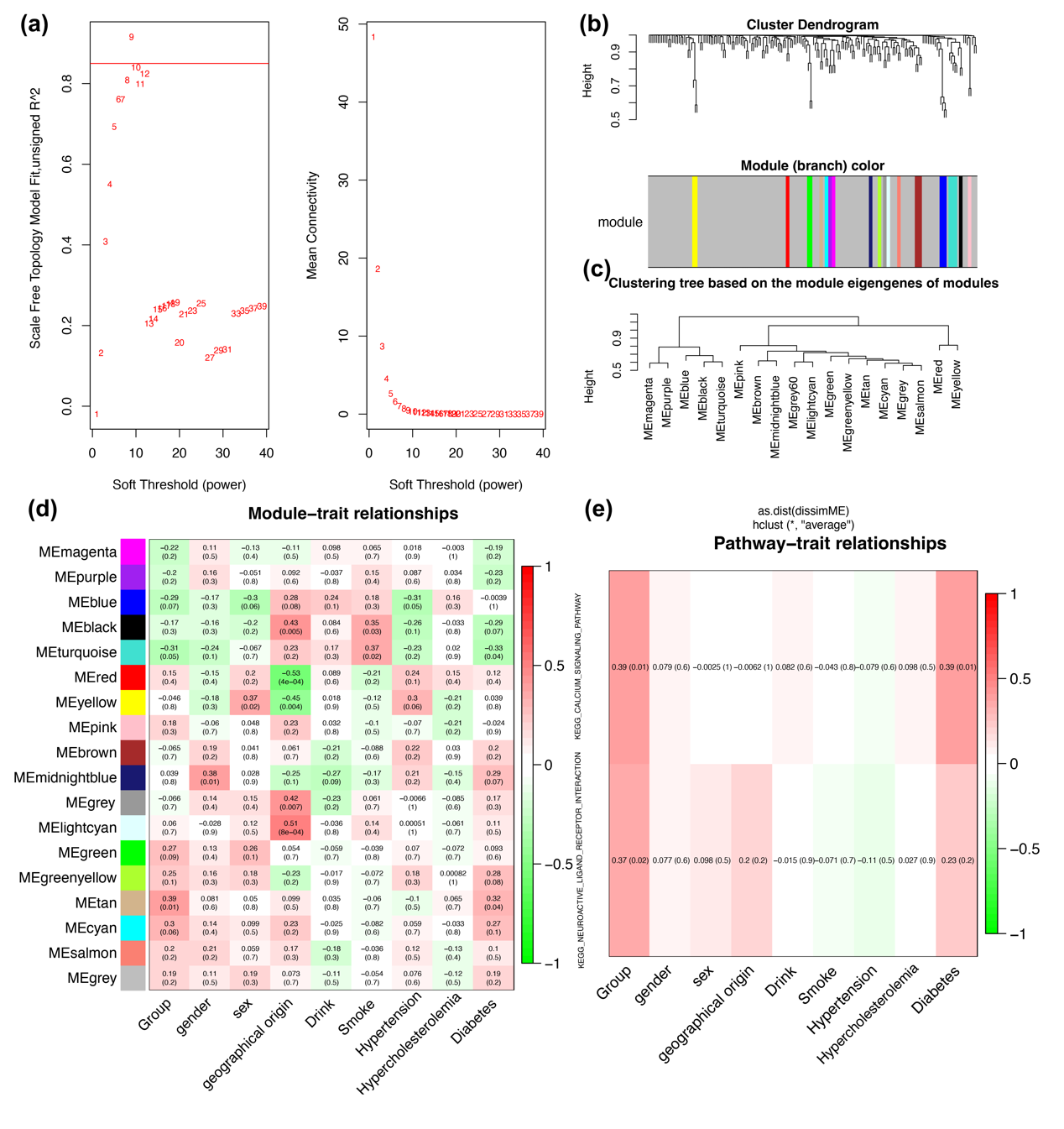


Supplementary Figure 1: Pathway modules derived from WGCNA based on the KEGG pathway analysis of GSE22255.

(a) Analysis of a scale-free fit index for various soft thresholding powers.

(b) Number of pathway clusters and module cuts.

(c) Clustering of each pathway module.

(d) A heat map of correlation analysis between modules and patient phenotypes.

(e) A heat map of correlation analysis between the KEGG pathways in the tan module and patient phenotypes.


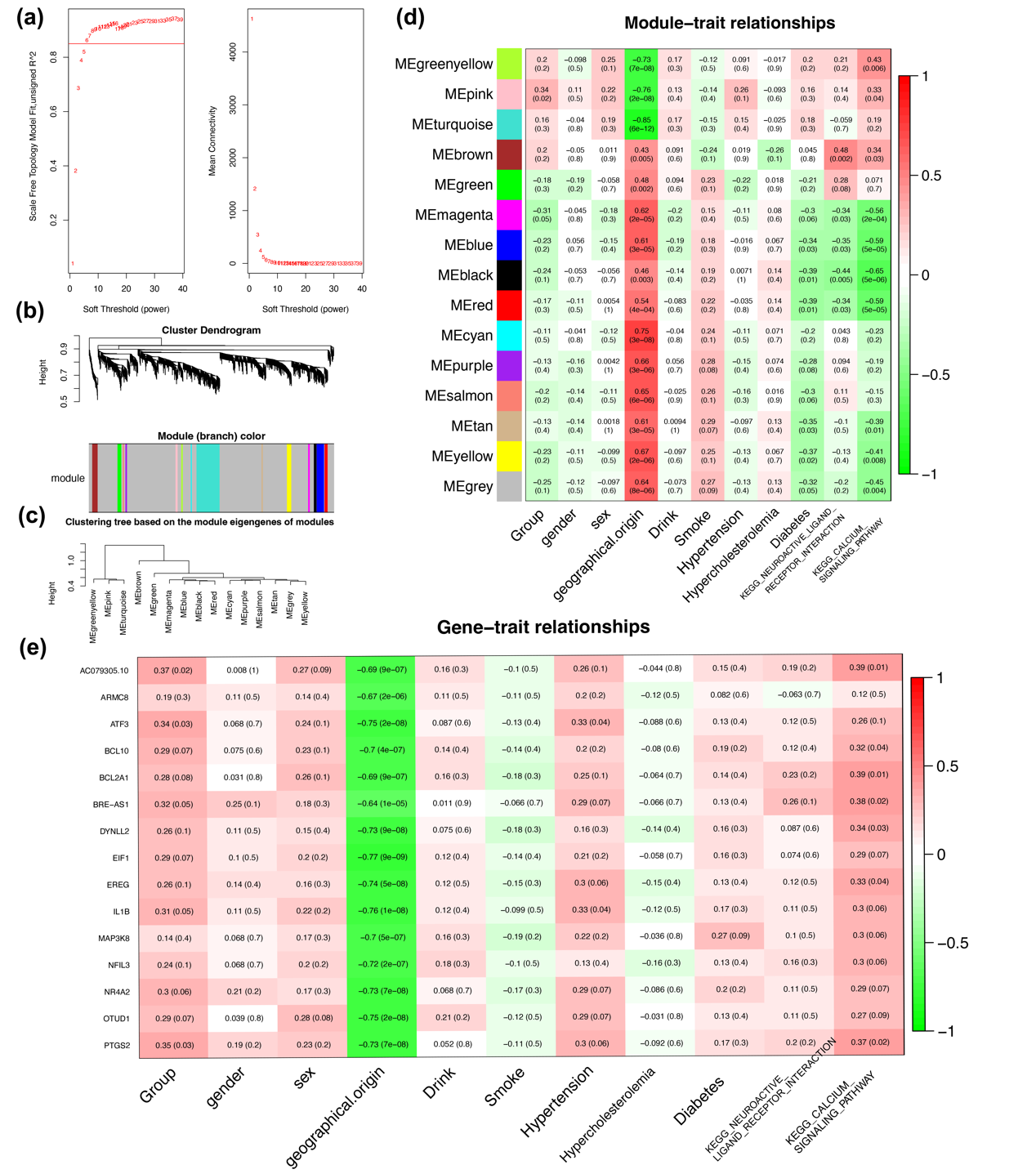


Supplementary Figure 2: Gene modules based on the gene expression of GSE22255.

(a) Analysis of a scale-free fit index for various soft thresholding powers.

(b) Number of gene clusters and module cuts.

(c) Clustering of each gene module.

(d) A heat map of correlation analysis between modules and patient phenotypes.

(e) A heat map of correlation analysis between the genes in the pink module and patient phenotypes.


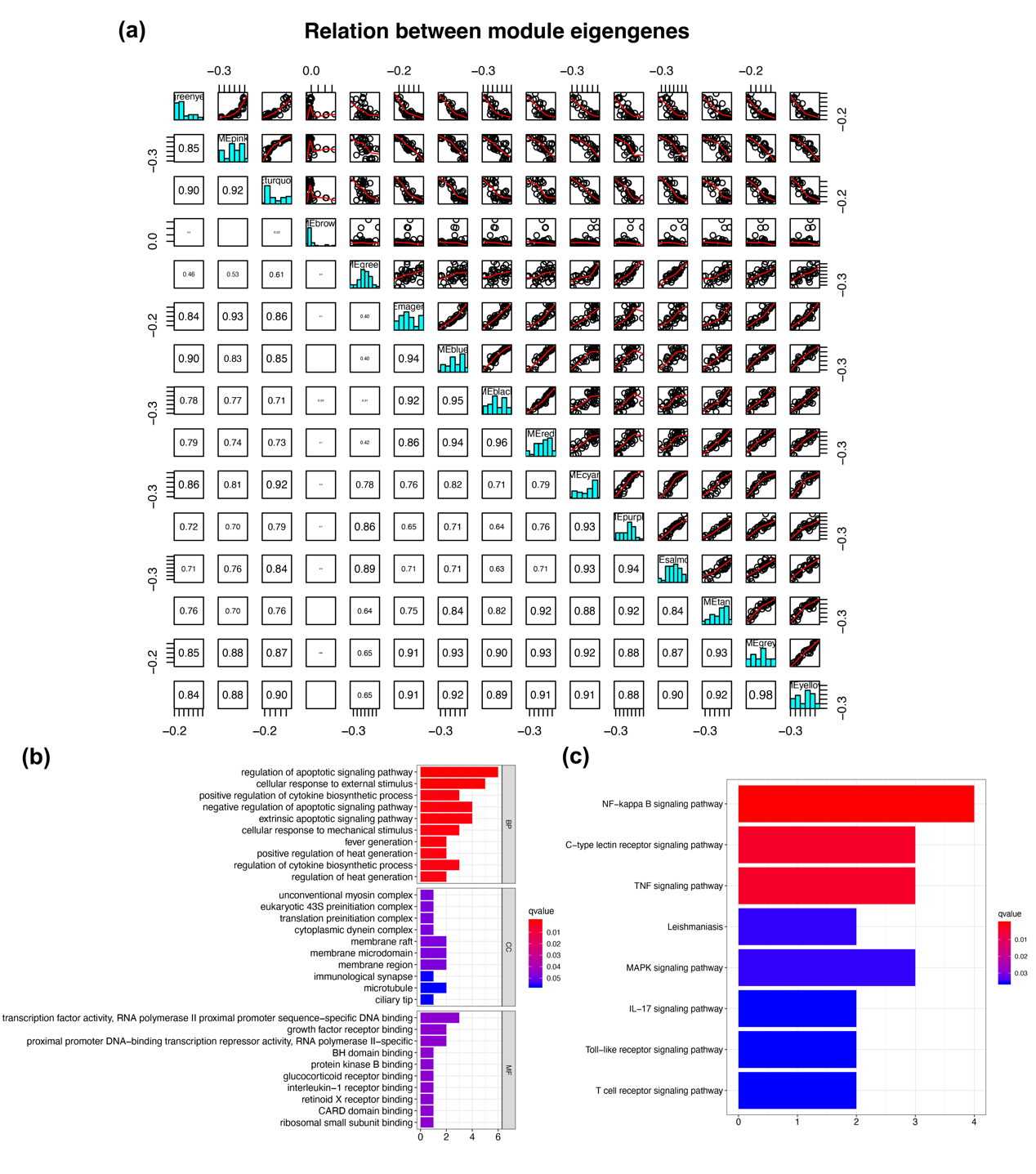


Supplementary Figure 3: Relationship among gene module eigengenes and enrichment analyses of the calcium signaling pathway-related genes.

(a) Correlation analysis among gene module eigengenes showed a strong correlation between the pink module and other modules except for the brown module.

(b) GO enrichment analysis.

(c) KEGG enrichment analysis.


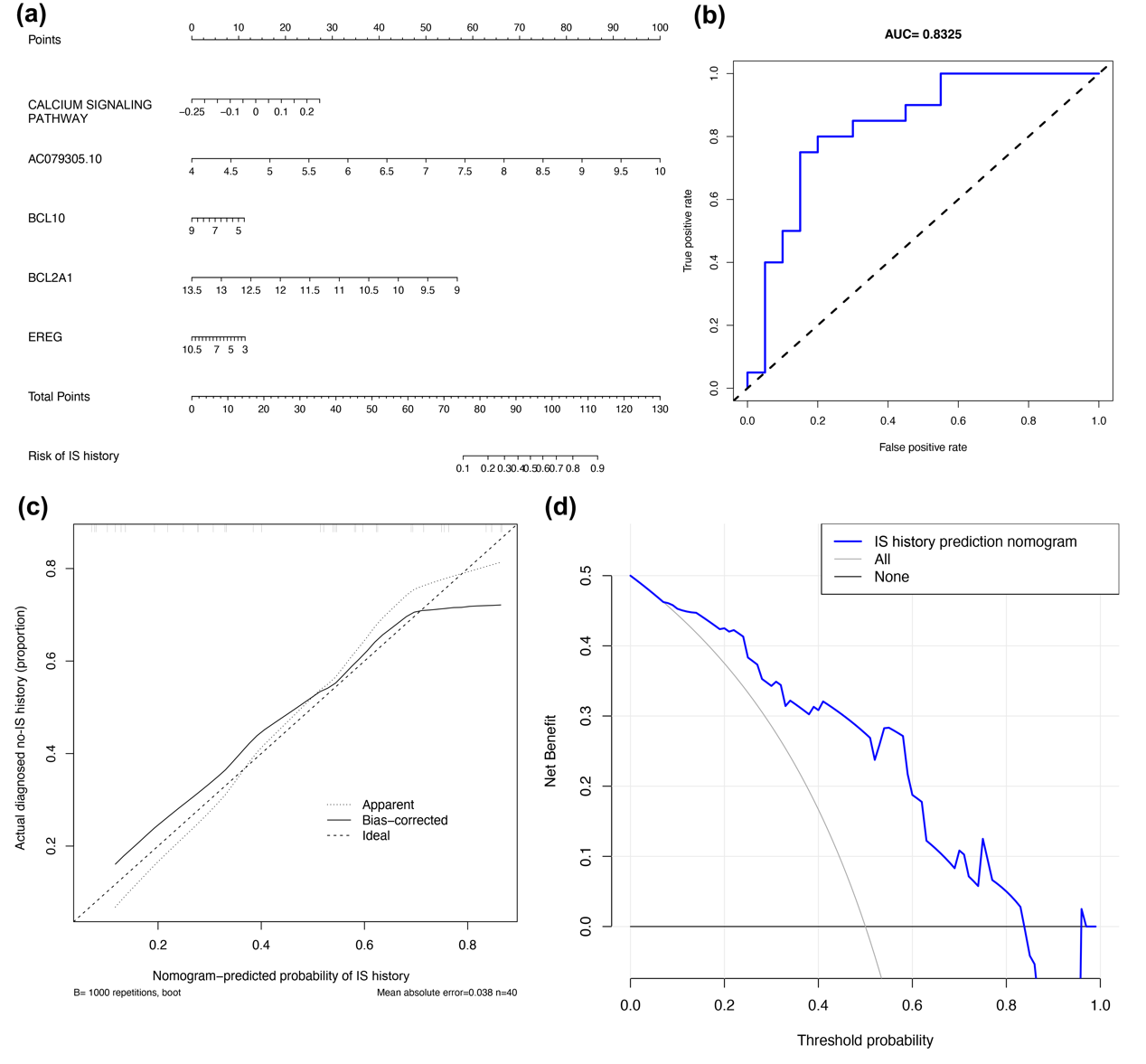


Supplementary Figure 4: Constructing a predictive model of IS history.

(a) Nomogram prediction model of IS history.

(b) ROC curve for the predictive model of IS history (AUC = 0.8325).

(c) Calibration curve for the predictive model of IS history.

(d) Decision curve showing the probability of benefit from an intervention based on this predictive model.


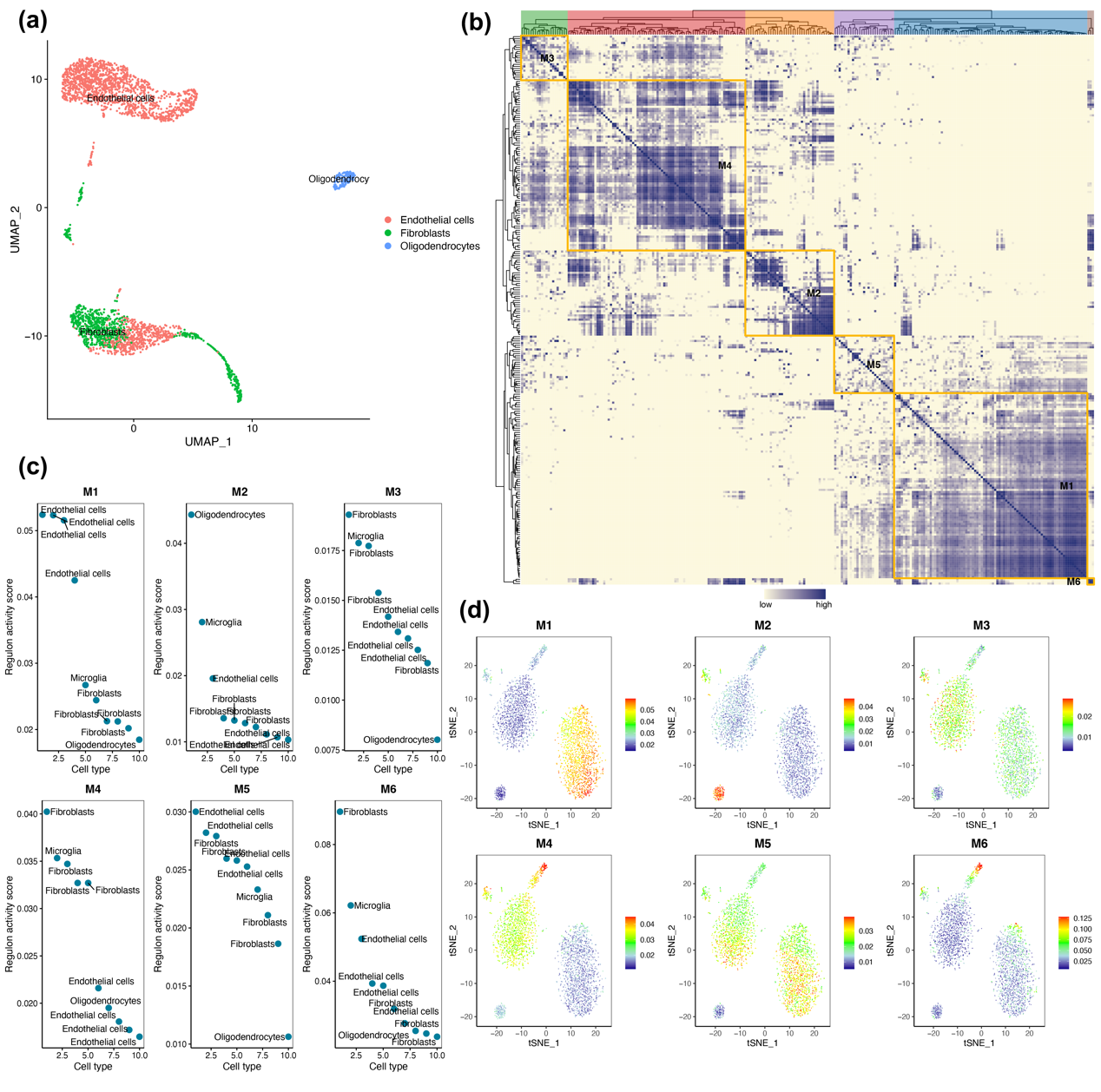


Supplementary Figure 5: Identification of regulon modules based on the regulation CSI matrix in mouse brain vasculature cells.

(a) Annotation of cell clusters using SingleR.

(b) Determination of the regulon modules.

(c) Ranks for cell types based on regulon specificity scores.

(d) Average activity of each module.


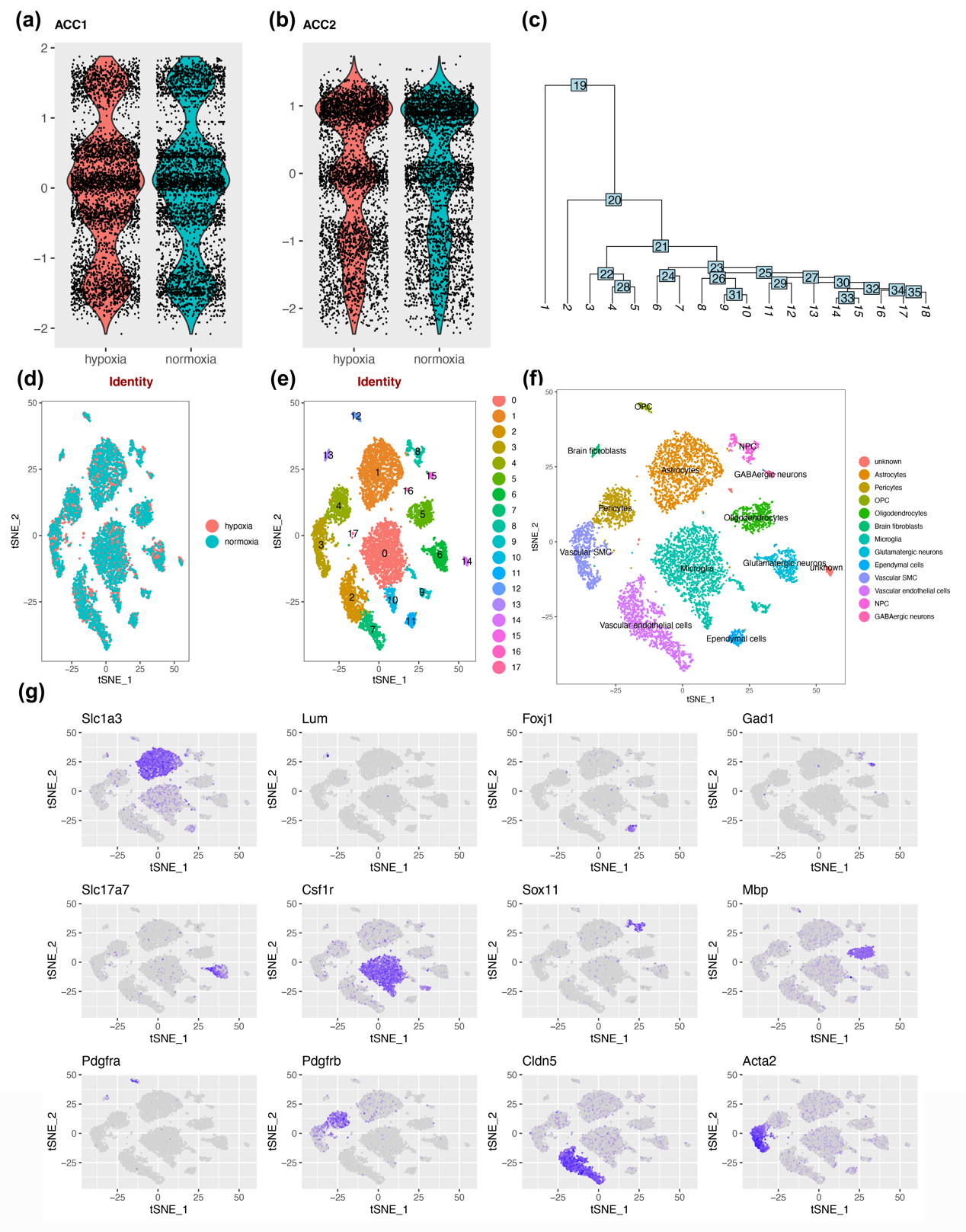


Supplementary Figure 6: Integration, dimensionality reduction and cluster annotation of scRNA-seq data in the mouse cerebral cortex.

(a–b) Visualisation of the aligned CCA in hypoxia and normoxia.

(c) A phylogenetic tree based on the distance matrix in gene vascular endothelial cells vascular endothelial cells vascular endothelial cells expression space computed to assess the robustness of the clusters.

(d–e) Integrated analysis of the two cell groups.

(f) Annotation of cell clusters with marker genes reported in the original study.

(g) Visualisation of the marker genes in tSNE plots.


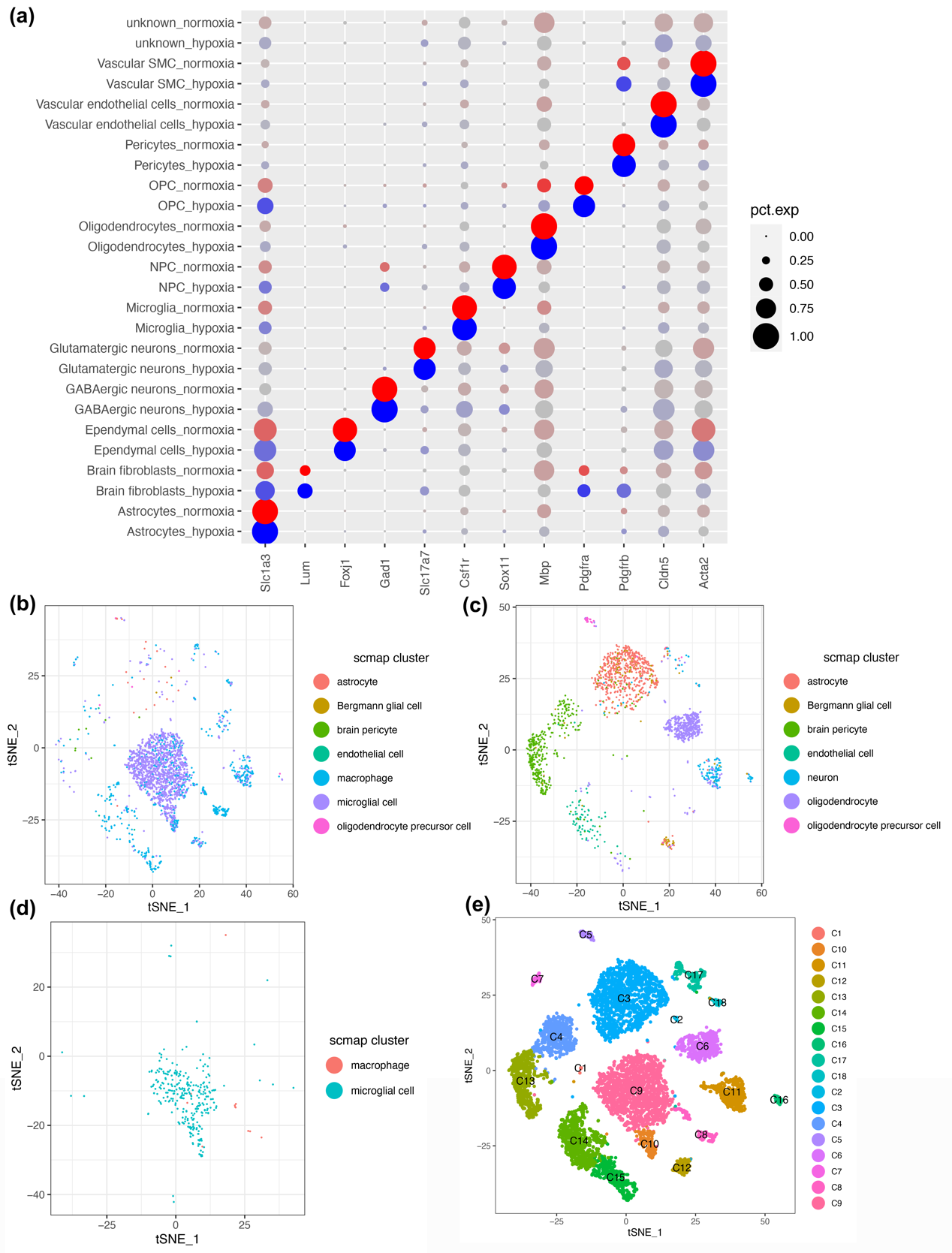


Supplementary Figure 7: Annotation of scRNA-seq data in the mouse cerebral cortex using scmap.

(a) Comparison of marker genes expression between the hypoxia and normoxia groups.

(b–d) Annotation of clusters using scmap.

(e) Visualisation of clustering results.


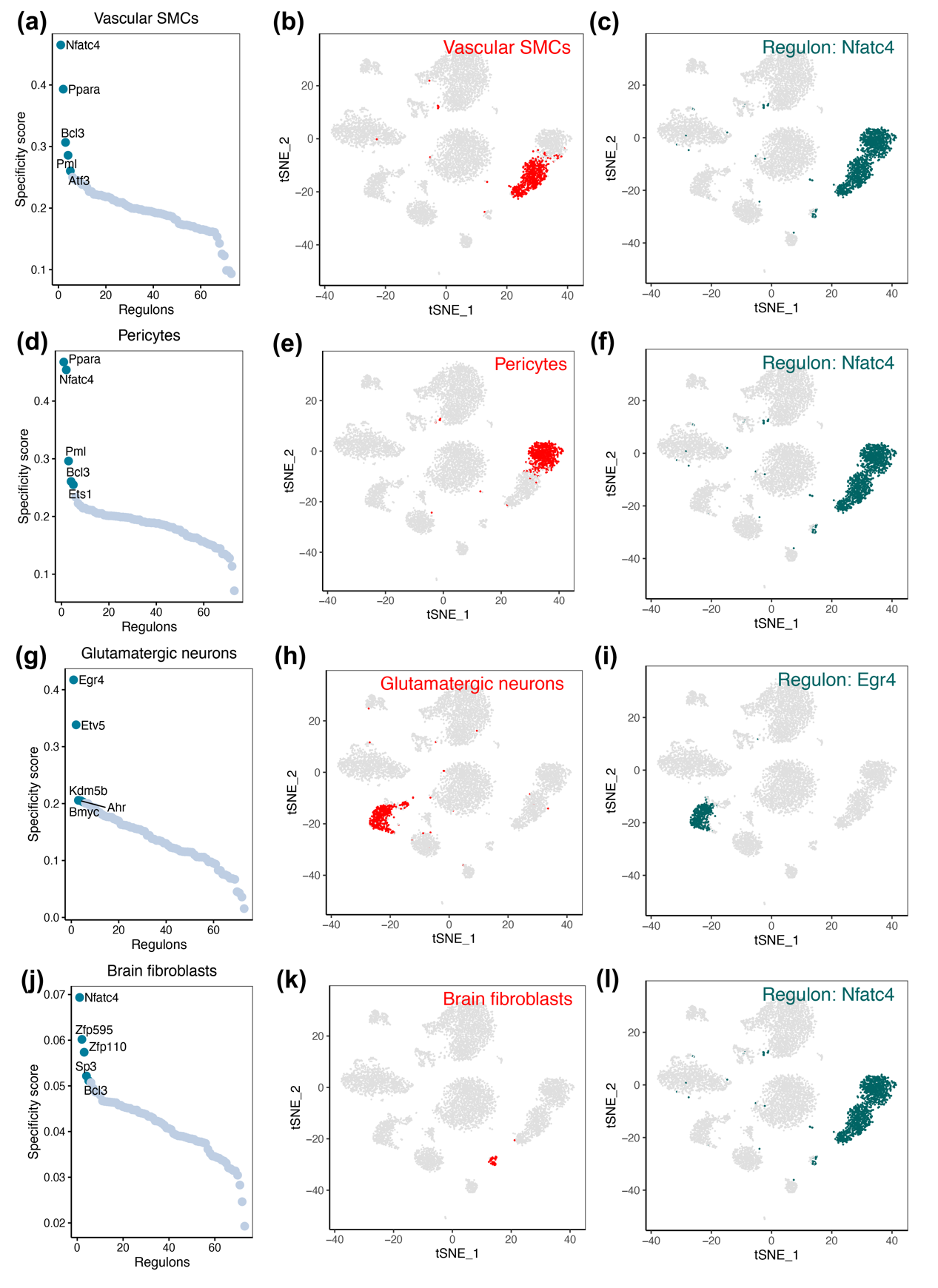


Supplementary Figure 8: Cell type-specific regulons in the mouse cerebral cortex.

(a) Ranks of regulons in mouse cerebral cortex vascular SMCs sorted based on regulon specificity scores (right) and the corresponding binding motifs of TFs.

(b) Vascular SMCs are highlighted as red dots in the tSNE plot.

(c) The expression values of interesting genes are presented as green dots in the tSNE plot.

(d–f) Same as (a–c) but for pericytes.

(g–i) Same as (a–c) but for glutamatergic neurons.

(j–l) Same as (a–c) but for brain fibroblasts.


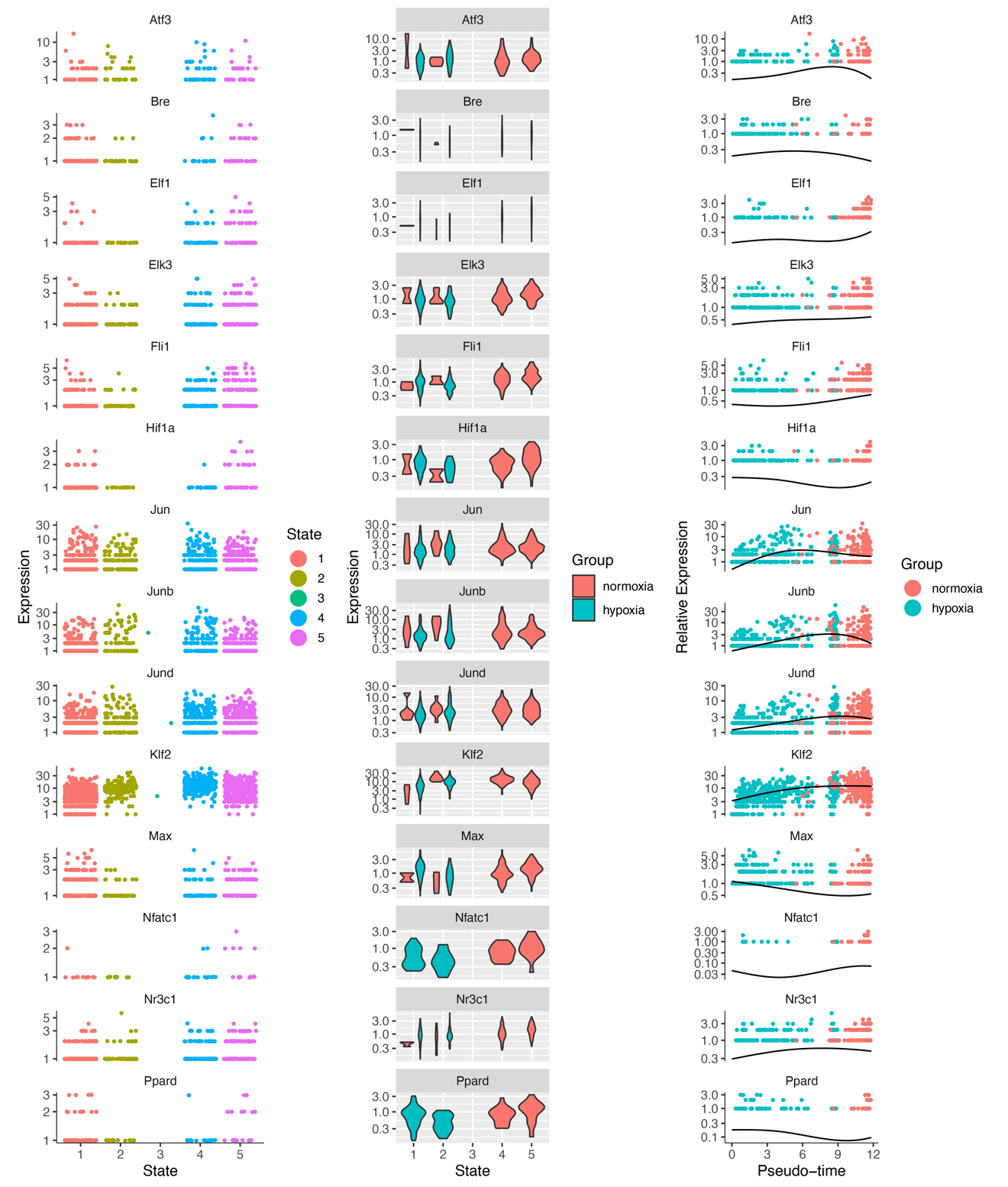


Supplementary Figure 9: Differential expression of pseudotime-related molecules in the states and between the two groups.


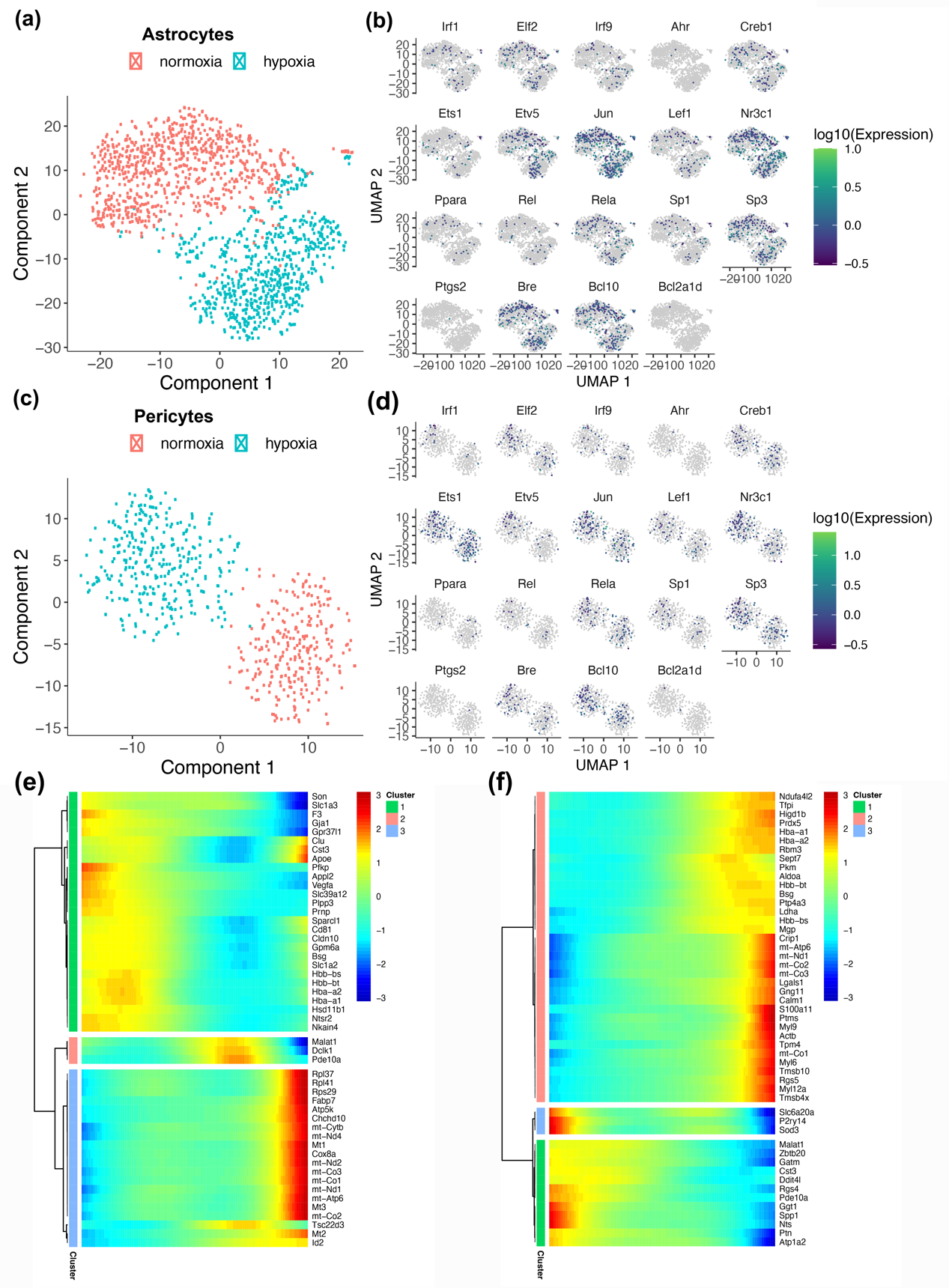


Supplementary Figure 10: UMAP plot of mouse cerebral cortex cells.

(a) UMAP plot of astrocytes in normoxia and hypoxia.

(b) Expression of the 19 hub TFs and target genes in astrocytes.

(c–d) Same as (a–b) but for pericytes.

(e) A heat map of pseudotime-related genes in astrocytes.

(f) A heat map of pseudotime-related genes in pericytes.


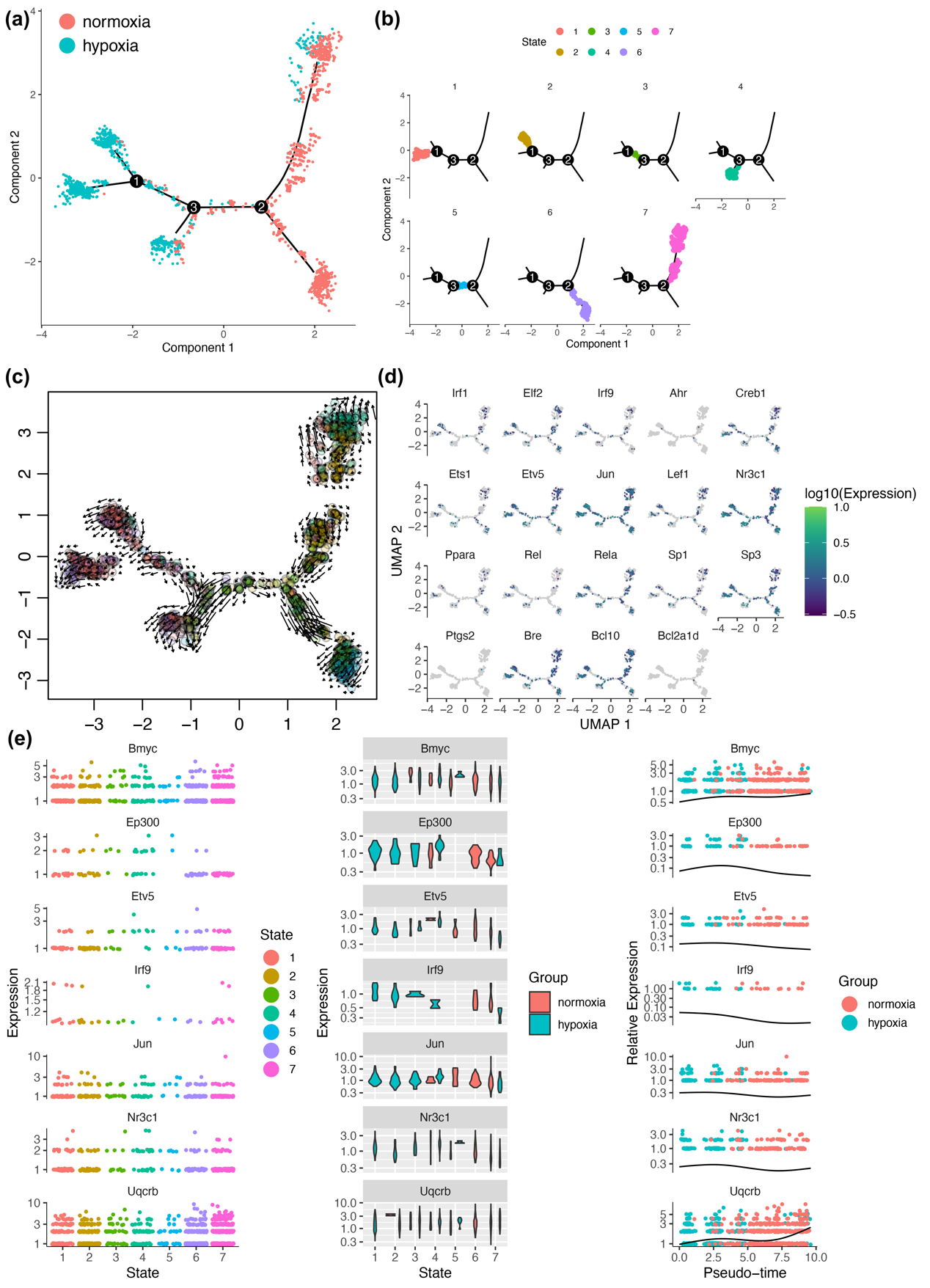


Supplementary Figure 11: Pseudotemporal and RNA velocity analyses of astrocytes.

(a) DDRTree plot of astrocytes in normoxia and hypoxia.

(b) State distribution of astrocytes in the DDRTree plot.

(c) RNA velocity plot with longer arrows representing stronger transcriptional activity.

(d) Expression of 19 hub TFs and target genes.

(e) Expression and pseudotime of pseudotemporal-related hub TFs.


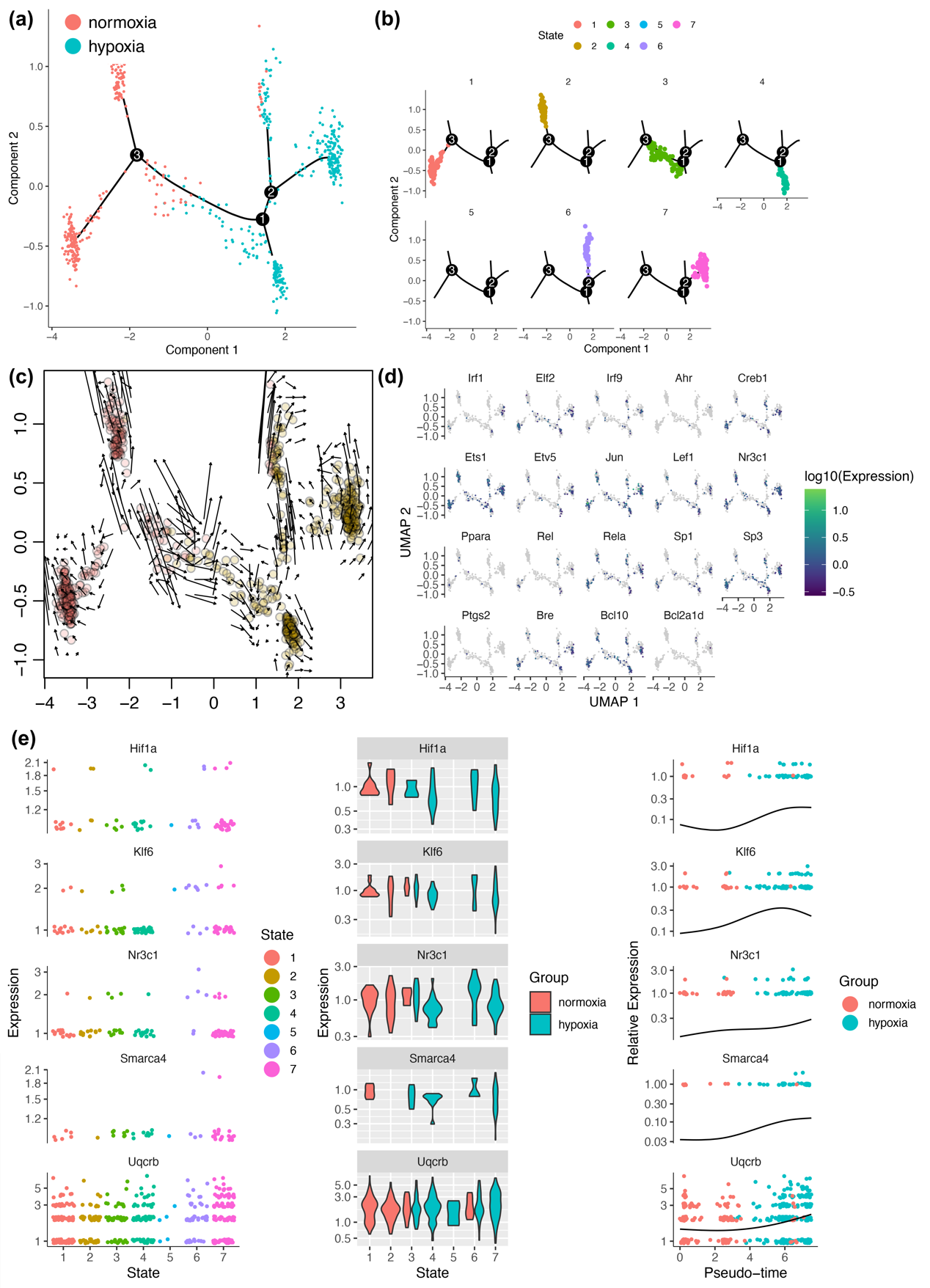


Supplementary Figure 12: Pseudotemporal and RNA velocity analyses of pericytes.

(a) DDRTree plot of pericytes in normoxia and hypoxia.

(b) State distribution of pericytes in the DDRTree plot.

(c) RNA velocity plot with longer arrows representing stronger transcriptional activity.

(d) Expression of 19 hub TFs and target genes.

(e) Expression and pseudotime of pseudotemporal-related hub TFs.

**Supplementary Tables**

| Supplementary table 1. Demographic characteristics of GSE22255 | | | | |
| --- | --- | --- | --- | --- |
| Variables | Control | IS | Total | *p* |
|  | (n=20) | (n=20) |  |  |
| Age | 58.70±10.99 | 60.20±10.57 | 59.45±10.67 | 0.662 |
| Gender |  |  |  |  |
| Female | 10(50.00) | 10(50.00) | 20(50.00) | 1 |
| Male | 10(50.00) | 10(50.00) | 20(50.00) |  |
| Geographical origin |  |  |  |  |
| Lisboa | 5(25.00) | 0(0.00) | 5(12.50) | 0.117 |
| Mirandela | 7(35.00) | 8(40.00) | 15(37.50) |  |
| Porto | 3(15.00) | 5(25.00) | 8(20.00) |  |
| Vila-Real | 5(25.00) | 7(35.00) | 12(30.00) |  |
| Drink |  |  |  |  |
| NO | 12(60.00) | 7(35.00) | 19(47.50) | 0.113 |
| YES | 8(40.00) | 13(65.00) | 21(52.50) |  |
| Smoke |  |  |  |  |
| NO | 14(70.00) | 15(75.00) | 29(72.50) | 0.723 |
| YES | 6(30.00) | 5(25.00) | 11(27.50) |  |
| Hypertension |  |  |  |  |
| NO | 12(60.00) | 8(40.00) | 20(50.00) | 0.206 |
| YES | 8(40.00) | 12(60.00) | 20(50.00) |  |
| Hypercholesterolemia |  |  |  |  |
| NO | 13(65.00) | 10(50.00) | 23(57.50) | 0.337 |
| YES | 7(35.00) | 10(50.00) | 17(42.50) |  |
| Diabetes |  |  |  |  |
| NO | 20(100.00) | 16(80.00) | 36(90.00) | 0.035* |
| YES | 0(0.00) | 4(20.00) | 4(10.00) |  |
| * p<0.05 ** p<0.01 |  |  |  |  |

| Supplementary table 2. Univariable logistic regression analysis | | | |
| --- | --- | --- | --- |
| Variables | *β* | Univariable logistic regression analysis Odd ratio (95% CI) | *p* |
|  |  |  |  |
| Gender | 0.00 | 1(0.29-3.49) 3.49 | 1.00 |
| Age | 0.01 | 1.01(0.96-1.08) | 0.65 |
| Geographical.origin | -0.03 | 0.94(0.57-1.54) | 0.80 |
| Drink | 0.44 | 2.79(0.79-10.52) | 0.12 |
| Smoke | -0.11 | 0.78(0.19-3.15) | 0.72 |
| Hypertension | 0.35 | 2.25(0.65-8.28) | 0.21 |
| Hypercholesterolemia | 0.27 | 1.86(0.53-6.84) | 0.34 |
| Diabetes | 7.73 | 53181015.43(0-NA) | 0.99 |
| NEUROACTIVE LIGAND RECEPTOR INTERACTION | 2.62 | 413.29(2.75-150766.39) | 0.03 |
| CALCIUM SIGNALING PATHWAY | 2.69 | 491.33(3.59-161197.27) | 0.02 |
| AC079305.10 | 0.22 | 1.67(1.1-2.75) | 0.03 |
| BCL10 | 0.23 | 1.71(0.98-3.22) | 0.07 |
| BCL2A1 | 0.22 | 1.67(0.95-3.14) | 0.09 |
| BRE.AS1 | 0.14 | 1.37(1.01-1.98) | 0.06 |
| DYNLL2 | 0.38 | 2.38(0.86-7.32) | 0.11 |
| EREG | 0.11 | 1.28(0.96-1.76) | 0.11 |
| Note: *β* is regression coefficient | | | |

| Supplementary table 3. Multivariable logistic regression analysis | | | |
| --- | --- | --- | --- |
| Variables | *β* | Multivariable logistic regression analysis Odd ratio (95% CI) | *p* |
|  |  |  |  |
| (Intercept) | 1374.73 | 1374.73(0-NA) | 0.41 |
| CALCIUM SIGNALING PATHWAY | 620.26 | 620.26(1.51-777195.61) | 0.05 |
| AC079305.10 | 7.12 | 7.12(1.26-55.87) | 0.04 |
| BCL10 | 0.75 | 0.75(0.11-4.27) | 0.75 |
| BCL2A1 | 0.23 | 0.23(0.02-2.16) | 0.21 |
| EREG | 0.84 | 0.84(0.26-2.48) | 0.75 |
| Note: *β* is regression coefficient | | | |
